# Smart building energy management with renewables and storage systems using a modified weighted mean of vectors algorithm

**DOI:** 10.1038/s41598-024-79782-5

**Published:** 2025-02-08

**Authors:** Mohamed Ebeed, Sabreen hassan, Salah Kamel, Loai Nasrat, Ali Wagdy Mohamed, Abdel-Raheem Youssef

**Affiliations:** 1 Department of Electrical Engineering, Faculty of Engineering, Sohag University, Sohag, 82524 Egypt; 2 Department of Electrical Engineering, Aswan University, Aswan, 81542 Egypt; 3 Operations Research Department, Faculty of Graduate Studies for Statistical Research, Cairo University, Giza, 12613 Egypt; 4 Department of Electrical Engineering, Faculty of Engineering, South Valley University, Qena, 83523 Egypt

**Keywords:** Smart home, Weighted mean of vectors algorithm, Cost, Peak to average ratio, Demand side response, Engineering, Mathematics and computing

## Abstract

With the advancement of automation technologies in household appliances, the flexibility of smart home energy management (EM) systems has increased. However, this progress has brought about a new challenge for smart homes: the EM has become more complex with the integration of multiple conventional, renewable, and energy storage systems. To address this challenge, a novel modified Weighted Mean of Vectors algorithm (MINFO) is proposed. This algorithm aims to enhance the performance of smart building EM by overcoming the limitations of conventional approaches, such as low solution accuracy and inadequacy in handling complex problems. MINFO operates on two key principles. Firstly, it employs the Elite Centroid Quasi-Oppositional Base Learning (ECQOBL) approach to improve the exploitation capabilities of conventional algorithms. Secondly, it utilizes an Adaptive Levy Flight Motion (ALFM) technique to enhance exploration. The EM problem tackled involves optimizing the scheduling of multiple energy sources, including diesel generators, PV units, and batteries, within a smart building context. Additionally, it incorporates time-of-use-based demand-side response (DSR) to manage shiftable loads, thereby reducing electricity costs and peak-to-average ratio (PAR) simultaneously and independently. The effectiveness of MINFO is demonstrated through comprehensive evaluations, comparing its performance with other optimization methods across 33 benchmark functions from basic and CEC-2019 test suites. Results indicate that MINFO significantly improves smart building EM, achieving a reduction of 53.20% in electricity costs (cost only), 53.19% in PAR (PAR only), and 50.84% in combined cost and PAR compared to the base case. These findings underscore the robustness of MINFO as an optimizer for smart building energy management.

## Introduction

Currently, there has been a significant rise in energy consumption, with global usage escalating from 13,277 billion kWh in 2000 to 22,347 billion kWh by 2017, as illustrated by data from the U.S. Energy Information Administration (EIA)^1^. Furthermore, the residential loads demand represents about 13–37% of the total loads demand^2^. Thus, it is mandatory solving the energy management of the residential buildings to maximize the utilization of the existing energy sources to cove the growth in the load demand.

**Fig. 1:**
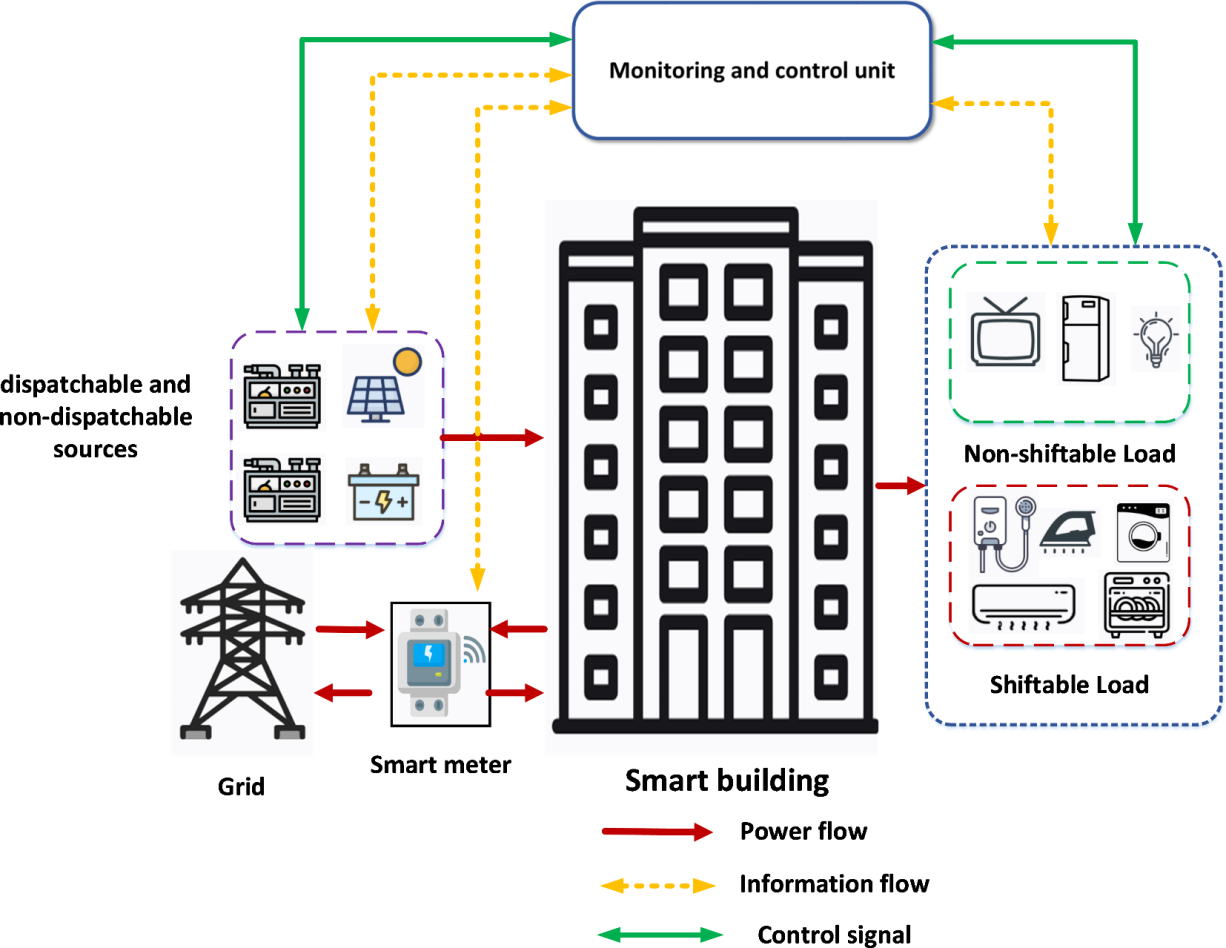
The structure of smart home.

The recent trend of the building sectors is to shift away from the conventional home to smart home automation in which it can provide several benefits like ability to control the energy consumption which provide the stability and ability of the electric system. The infrastructure of the smart home is depicted in Fig. 1. The aim of the smart home is to monitor and control the consumption of the appliances. Thus, the smart homes system has a two-way communication system and the smart metering unit. The loads of the smart homes can be categorized as shiftable (transferable) loads and non-shiftable loads. Furthermore, the smart home has an interface system, and the control system.

The optimal energy management of the smart building means obtain the best consumption of the load demand and the optimal scheduling coordination of the interconnected energy sources like diesel generators, PV units, wind turbines and other renewable energy sources^3,4^. The load management can be accomplished by application the demand side response (DSR) program which mainly depends upon different strategies like real-time pricing (RTP), critical peak pricing (CPP), Time of use (TOU) and extreme day pricing (EDP)^5–8^.

The EM of the smart homes was solved by different optimization methods where in antlion optimization algorithm was employed for EM solution for multi-objective functions including the cost, the PAR and the user comfort with CPP and RTP based DSR^9^. The authors in^10^ solved EM of the smart home using a modified MRFO for PAR and cost minimization with maximum utilization of the PV panel, wind turbine and batteries. The game theory method was implemented for EM of smart home for bill energy and PAR reduction in presence of wind turbine and PV units. The community-to-community strategy was proposed for the EM the electricity bill and PAR minimization^11^. The EM of multi homes was solved using artificial bee colony to minimize the cost of energy based on market clearing price (MCP)^12^. The genetic harmony search algorithm was implemented for EM of smart home to reduce the PAR and electricity bill with taken into consideration the RTP and CPP^13^. The elite evolutionary strategy artificial ecosystem optimization was presented for smart home EM solution for energy bill and PAR reduction and customer comfort maximization considering RTP, CCP and TOU tariffs^14^. A hybrid particle swarm optimization and harmony search algorithm were implemented for EM solution for the bill cost minimization in the presence of Electric vehicles (EVs)^15^. An improved bald eagle search algorithm for energy bill and PAR reduction and customer comfort maximization^16^. In^17^, the bacterial foraging ant colony optimization was presented to solve the EM of smart home for reducing RTP and the energy bill cost considering CPP and TOU. In^18^, the EM of smart home was solved with inclusion of renewable energy system, storage units and EVs for reducing the cost and the net present cost (NPC).

INFO^19^ is one of the newest efficient optimizers that was employed for solving many optimization problem like the power system stabilizer design^20^, parameters of the solar cell^21^, disease classification^22^, the optimal power flow^23^, designing a hybrid energy system^24^ and optimization the proportional-integral-derivative (PID). According to the comprehensive survey, the paper’s contribution to the field of smart building energy management, compared to other existing optimization methods, lies in its approach to not only apply Demand Side Response (DSR) for appliances but also provide an optimal scheduling of energy sources to significantly reduce costs. The main challenge in smart home energy management is optimizing demand response while considering multiple energy sources, such as PV, diesel generators, and batteries, which increases the complexity of the problem. It is important to note that the traditional INFO technique tends to suffer from stagnation and becomes trapped in local optima when applied to highly nonlinear optimization problems. To address these limitations, the MINFO algorithm is proposed, incorporating three improvement strategies to enhance both the exploration and exploitation capabilities of the optimizer. In this regard two improvement approaches are applied including the ECQOBL and ALFM to improving its performance and searching abilities. The main contributions of this work are depicted below.

- A modified version of the Weighted mean of vectors algorithm (MINFO) is developed to solve the EM of a smart building for solving the EM of the smart building under three cases including cost reduction only, (2) PAR reduction only, and cost with PAR minimization simultaneously.
- The MINFO is based on two novel modifications including an elite centroid quasi-oppositional base learning (ECQOBL) and an adaptive levy flight motion (ALFM).
- The performance of the MINFO is tested on 23 basic benchmark functions and 10 CEC-2019 benchmark functions.
- The robustness of the MINFO is evaluated based of the statistical analysis, qualitative analysis, convergence trends, and boxplot analysis.
- The obtained results by MINFO for all studied cases are compared with other 9 optimization techniques including basic INFO^19^, sand cat search optimizer (SCSO)^25^, African vultures optimization algorithm (AVOA)^26^, gery wolf optimizer (GWO)^27^, Harris hawks optimization (HHO)^28^, zebra optimization algorithm (ZOA)^29^, whale optimization algorithm (WOA)^30^, RIME algorithm^31^ and Dung beetle optimize (BDO)^32^.

The rest of paper is organized in 4 sections in which ‘Problem definition’ section describes the required objective functions to be optimized and the associated constraints. Sections ‘INFO’ and ‘MINFO’ explain the concepts and mathematical equations of the proposed optimizers. Section ‘Simulation and results’ presents and discuss the obtained results by application the MINFO and other techniques on the benchmark function and for the EM solution. Finally, the ‘Conclusion’ section outlines the main findings of this works.

## Problem definition

The aim of the energy management of the smart building is to reduce total cost as well as to minimize the peak-to-average ratio ($\:PAR$). It should be highlighted here that the load demand of the smart building can be divided into shiftable loads ($\:{P}_{SH}$) and non-shiftable loads ($\:{P}_{NSH}$) which can be represented as follows:

1 $$\:{P}_{Load1}={P}_{SH}=\left[{P}_{SH}^{1},{P}_{SH}^{2},\dots\:,{P}_{SH}^{T}\right]$$

2 $$\:{{P}_{Load2}=P}_{NSH}=\left[{P}_{NSH}^{1},{P}_{NSH}^{2},\dots\:,{P}_{NSH}^{T}\right]$$

3 $$\:{P}_{Ltotal}={P}_{SH}+{P}_{NSH}$$

where $\:{P}_{Ltotal}$ is the total load demand. $\:T$ represents total number of time slots. The total operating cost ($\:TCost$) including the electricity bill cost ($\:{Cost}_{bill}$), the operating cost of the PV units ($\:{Cost}_{PV}$), the operating cost of the battery units ($\:{Cost}_{battery}$) and the operating cost of the diesel generators ($\:{Cost}_{D}$) which can be represented as follows:

4 $$\:{F}_{1}=TCost=\:{Cost}_{bill}+\:{Cost}_{PV}+{Cost}_{D}+{Cost}_{Battery}$$

In which

5 $$\:Cost_{{bill}} \: = \sum {\:_{{t = 1}}^{T} } \left( {Cost_{{buy}}^{t} - Cost_{{sell}}^{t} } \right) = \sum {\:_{{t = 1}}^{{24}} } \left( {P_{{buy}}^{t} \times \:K_{{buy}}^{t} - P_{{sell}}^{t} \times \:K_{{sell}}^{t} } \right);\forall \:\:t \in T\:$$

6 $$\:{Cost}_{D}={K}^{D}\times\:\sum\:_{t}^{T}{P}_{D}^{t};\forall\:\:t \in T$$

7 $$\:{Cost}_{PV}={K}^{PV}\times\:\sum\:_{t}^{T}{P}_{PV}^{t}\:;\forall\:\:t \in T$$

8 $$\:{Cost}_{Battery}={K}^{Battery}\times\:\sum\:_{t}^{T}{P}_{Battery}^{t}\:;\forall\:\:t \in T$$

In which, the generated power during the 24-hour by the PV ($\:{P}_{t}^{PV}$) cab be calculated as follows^33^:

9 $$\:{P}_{PV}^{t}\:=\hspace{0.33em}\hspace{0.33em}\left\{\begin{array}{c}{P}_{r}\:\left(\frac{{\left({G}^{t}\right)}^{2}}{{G}_{std\:}\times\:{X}_{c}\:\:\:\:\:}\right)\:\:\:for\:\:0<{G}^{t}\le\:{X}_{c}\\\:{\:\:\:\:\:\:P}_{r}\left(\frac{{G}^{t}}{{G}_{std\:}\:}\right)\:\:\:\:\:\:\:\:\:\:\:for\:\:\:\:\:{G}^{t}\ge\:{X}_{c}\:\:\:\:\:\:\:\:\:\:\:\:\end{array}\right.;\forall\:\:t \in T\:$$

Where $\:{P}_{r}$ is its rated power. $\:{P}_{buy}^{t}$ and $\:{P}_{sell}^{t}$ are the procurement and the sold powers to the grid at time segment, respectively. The second objective function is minimization the $\:PAR$ which can be expressed as follows^34^:

10 $$F_{2} = PAR = \frac{{max.\left( {P_{{Ltotal}} } \right)}}{{\sum {P_{{{\raise0.5ex\hbox{$\scriptstyle {Ltotal}$} \kern-0.1em/\kern-0.15em \lower0.25ex\hbox{$\scriptstyle T$}}}} } }}$$

The third objective function is multi-objective function to minimize the $\:TCost$ and the $\:PAR$ simultaneously as follows:

11 $$\:{F}_{3}={{\upomega\:}}_{1}\times\:\frac{TCost}{{TCost}_{base}}+{{\upomega\:}}_{2}\times\:\frac{PAR}{{PAR}_{base}}\:$$

$\:{TCost}_{base}$ and $\:{PAR}_{base}$ are the total operating cost and $\:PAR$ value in base case, i.e., without application the smart scheduling. $\:{{\upomega\:}}_{1}$ and $\:{{\upomega\:}}_{2}$ are weight factors in which each of them is selected to be 0.5. A set of constraints should be considered with solving the EM of the smart home including the equality and inequality operator limits which can be represented as follows:

12 $$\:{P}_{PV}^{t}+{P}_{D1}^{t}+{P}_{D2}^{t}+{P}_{Battery}^{t}+{P}_{Grid}^{t}={P}_{Load}^{t}$$

13 $$\:{E}^{t}=\:{E}^{t-1}+{\xi\:}_{ch}\:\times\:{P}_{ch}^{t}-\frac{1}{{\xi\:}_{dis}}{P}_{dis}^{t}\:$$

14 $$\:\underset{\_}{{P}_{r}}\le\:{P}_{r}\le\:\stackrel{-}{{P}_{r}}$$

15 $$\:\underset{\_}{{P}_{Grid}^{t}}\le\:{P}_{Grid}^{t}\le\:\stackrel{-}{{P}_{Grid}^{t}}\:;\forall\:\:t \in T$$

16 $$\:\underset{\_}{{P}_{D}^{t}}\le\:{P}_{D}^{t}\le\:\stackrel{-}{{P}_{D}^{t}}\:;\forall\:\:t \in T$$

17 $$\:\underset{\_}{{P}_{ch}}\le\:{P}_{ch}\le\:\stackrel{-}{{P}_{ch}}\:;\forall\:\:t \in T$$

18 $$\:\underset{\_}{{P}_{dis}}\le\:{P}_{dis}\le\:\stackrel{-}{{P}_{dis}}\:;\forall\:\:t \in T$$

where $\:\underset{\_}{\text{*}}$ and $\:\stackrel{-}{\text{*}}$ scripts refer to the minimum and maximum limits, respectively. $\:{P}_{dis}$ and $\:{P}_{ch}$ are the discharging and charging powers of the batteries, respectively.

## Weighted mean of vectors algorithm (INFO)

INFO is a novel optimization method that is deduced form a modified weight mean approach to update the positions of some vectors based on three steps including a local search, vector combining, and updating rule^19^. Weighted mean ($\:WM$) of two vectors can be formulated as follows:

19 $$\:WM=\frac{{x}_{1}\times\:{\tau\:}_{1}+{x}_{2}\times\:{\tau\:}_{2}}{{\tau\:}_{1}+{\tau\:}_{2}}$$

where $\:{\tau\:}_{1}$ and $\:{\tau\:}_{2}$ are the weight vectors which have been calculated using the wavelet function (WF) as follows:

20 $$\:\tau\:=\text{c}\text{o}\text{s}\left(x\right)\times\:\text{e}\text{x}\text{p}\left(-\frac{{x}^{2}}{\vartheta\:}\right)$$

where $\:\vartheta\:$ refers to the dilation parameter. As mentioned before the INFO is based on the three steps including updating rule, vector combining and Local search.

The steps of the INFO can be outlined as follows:

### Initialization

A set of populations can be generated randomly which can be represented as follows:

21 $$\:{X}_{i,j}=\left\{{X}_{i,1},{X}_{i,2},\dots\:,{X}_{i,D}\right\},i=\text{1,2},\dots\:,Np$$

where $\:Np$ and $\:D$ are number of populations and dimensions of the problem. It should be highlighted here that two important factors in INFO updating process which known as the scaling factor ($\:\sigma\:$) which has been utilized for scale the weighted mean of vectors while the another factor is the weighted mean factor ($\:\delta\:$) which has been used for amplifying the obtained vector.

#### Updating rule step

In this step, the weighted mean vectors are obtained from randomly selected differential vectors. the mean-based rule ($\:MR$) is employed for updating the

the vectors position which has been obtained from worst, better, and best solutions. The better solution can be selected randomly from the best 5 solutions. Hence, MR can be expressed as follows:

22 $$\:\text{MR\:}=r\times\:{MW1}_{i}+\left(1-r\right)\times\:{MW2}_{i}\:,\:i=\text{1,2},\dots\:,Np$$

In which

23 $$\:{MW1}_{i}=\:\delta\:\times\:\frac{{\tau\:}_{1}\left({x}_{a1}-{x}_{a2}\right)+{\tau\:}_{2}\left({x}_{a1}-{x}_{a3}\right)+{\tau\:}_{3}\left({x}_{a2}-{x}_{a3}\right)}{{\tau\:}_{1}+{\tau\:}_{2}+{\tau\:}_{3}+\epsilon\:}+\epsilon\:\times\:\text{\:rand,}i=\text{1,2},\dots\:,Np\text{\:}$$

24 $$\:{MW2}_{i}\:=\delta\:\times\:\frac{{W}_{1}\left({x}_{bs}-{x}_{bt}\right)+{W}_{2}\left({x}_{bs}-{x}_{ws}\right)+{W}_{3}\left({x}_{bt}-{x}_{ws}\right)}{{W}_{1}+{W}_{2}+{W}_{3}+\epsilon\:}+\epsilon\:\times\:rand,\:i=\text{1,2},\dots\:,Np$$

where $\:{\tau\:}_{1},{\tau\:}_{2}\:and\:{\tau\:}_{3}$ can be calculated as follows:

25 $$\:{\tau\:}_{1}=\text{c}\text{o}\text{s}\left(\left(f\left({x}_{a1}\right)-f\left({x}_{a2}\right)\right)+\pi\:\right)\times\:\text{e}\text{x}\text{p}\left(-\left|\frac{f\left({x}_{a1}\right)-f\left({x}_{a2}\right)}{\omega\:}\right|\right)$$

26 $$\:{\tau\:}_{2}=\text{c}\text{o}\text{s}\left(\left(f\left({x}_{a1}\right)-f\left({x}_{a3}\right)\right)+\pi\:\right)\times\:\text{e}\text{x}\text{p}\left(-\left|\frac{f\left({x}_{a1}\right)-f\left({x}_{a3}\right)}{\omega\:}\right|\right)$$

27 $$\:{\tau\:}_{3}=\text{c}\text{o}\text{s}\left(\left(f\left({x}_{a2}\right)-f\left({x}_{a3}\right)\right)+\pi\:\right)\times\:\text{e}\text{x}\text{p}\left(-\left|\frac{f\left({x}_{a2}\right)-f\left({x}_{a3}\right)}{\omega\:}\right|\right)$$

28 $$\:\tau\:=\text{m}\text{a}\text{x}\left(f\left({x}_{a1}\right),f\left({x}_{a2}\right),f\left({x}_{a3}\right)\right)$$

29 $$\:{W}_{1}=\text{c}\text{o}\text{s}\left(\left(f\left({x}_{bs}\right)-f\left({x}_{bt}\right)\right)+\pi\:\right)\times\:\text{e}\text{x}\text{p}\left(-\left|\frac{f\left({x}_{bs}\right)-f\left({x}_{bt}\right)}{\omega\:}\right|\right)$$

30 $$\:{W}_{2}=\text{c}\text{o}\text{s}\left(\left(f\left({x}_{bs}\right)-f\left({x}_{ws}\right)\right)+\pi\:\right)\times\:\text{e}\text{x}\text{p}\left(-\left|\frac{f\left({x}_{bs}\right)-f\left({x}_{ws}\right)}{\omega\:}\right|\right)$$

31 $$\:{W}_{3}=\text{c}\text{o}\text{s}\left(\left(f\left({x}_{bt}\right)-f\left({x}_{ws}\right)\right)+\pi\:\right)\times\:\text{e}\text{x}\text{p}\left(-\left|\frac{f\left({x}_{bt}\right)-f\left({x}_{ws}\right)}{\omega\:}\right|\right)$$

32 $$\:W=f\left({x}_{ws}\right)$$

where $\:f$ refers to the objective function. $\:\text{a}1\ne\:a2\ne\:a3\ne\:i$ refer to random integer values that were chosen from $\:[1,NP]$. $\:\epsilon\:$ and $\:\text{rand}$ denote a small value and a random value respectively. $\:{x}_{ws},{x}_{bt}$, $\:{x}_{bs}$ refers to are the worst, better, and best vectors, respectively. $\:r$ refers to a random value between the range of 0 and 0.5. The scaling factor can be calculated as follows:

33 $$\:\delta\:=2\beta\:\times\:\text{r}\text{a}\text{n}\text{d}-\beta\:$$

34 $$\:\beta\:=2\text{e}\text{x}\text{p}\left(-4\times\:\frac{t}{\text{\:}{t}_{\text{max\:}}}\right)$$

$\:{t}_{\text{max\:}}$ is the maximum number of iterations. In INFO, a convergence acceleration ($\:CA$) parameter was utilized for improving the global searching ability via helping the populations to move to the best direction. The *CA* is formulated as follows:

35 $$\:CA=\text{r}\text{a}\text{n}\text{d}\text{n}\times\:\frac{\left({x}_{bs}-{x}_{a1}\right)}{\left(f\left({x}_{bs}\right)-f\left({x}_{a1}\right)+\epsilon\:\right)}$$

In which *randn* reprents a random value that can be obtained from normal distribution. The new vector can be expressed as follows:

36 $$\:{x}_{new}=x+\sigma\:\times\:\text{MR\:}+CA$$

In which, the new two generated vectors can be obtained as described below.

37 $$\begin{gathered} if~rand<0.5 \hfill \\ {x_{new1}}=x+{\sigma _{sr}} \times {\text{MR}}+randndr \times \frac{{\left( {{x_{bs}} - {x_{a1}}} \right)}}{{\left( {f\left( {{x_{bs}}} \right) - f\left( {{x_{a1}}} \right)+1} \right)}} \hfill \\ else \hfill \\ {x_{new1}}={x_a}+{\sigma _{sr}} \times {\text{MR}}+randndr \times \frac{{\left( {{x_{a2}} - {x_{a3}}} \right)}}{{\left( {f\left( {{x_{a2}}} \right) - f\left( {{x_{a3}}} \right)+1} \right)}} \hfill \\ {x_{new2}}={x_{bt}}+{\sigma _{sr}} \times {\text{MR}}+randndr \times \frac{{\left( {{x_{a1}} - {x_{a2}}} \right)}}{{\left( {f\left( {{x_{a1}}} \right) - f\left( {{x_{a2}}} \right)+1} \right)}} \hfill \\ end \hfill \\ \end{gathered}$$

#### Vector combining step

For improving the diversity of the populations, the new generated vectors from the updating rule step are combined with the current vectors as follows:

$$\:\varvec{i}\varvec{f}\:randn<0.5\:\:\:$$

$$\:if\:randn<0.5$$

38.a $$\:u={x}_{new1}+\mu\:.|{x}_{new1}-{x}_{new2}|$$

$$\:else$$

38.b $$\:u={x}_{new1}+\mu\:.|{x}_{new1}-{x}_{new2}|$$

$$\:end$$

$$\:\varvec{e}\varvec{l}\varvec{s}\varvec{e}$$

38.c $$\:u=\:x$$

$$\:\varvec{e}\varvec{n}\varvec{d}$$

where u refers to the newly obtained solution. $\:\mu\:=0.5\times\:randn$.

#### Local search step

In this step the vectors are updated based on the best obtained solution which represents the exploitation stage of the INFO, and it can be described as follows:

39 $$\begin{gathered} f\;randn < 0.5 \hfill \\ if\;randn < 0.5 \hfill \\ u = x_{{bs}} + randn \times \left( {MR + randn \times \left( {x_{{bs}} - x_{{a1}} } \right)} \right) \hfill \\ \;else \hfill \\ u = x_{{randn}} + randn \times \left( {MR + randn \times \left( {m_{1} \times x_{{bs}} - m_{2} \times x_{{randn}} } \right)} \right) \hfill \\ end \hfill \\ \end{gathered}$$

40 $$\:{x}_{randn}=\rho\:\times\:{x}_{average}+\left(1-\rho\:\right)\times\:(\rho\:\times\:{x}_{bt}+(1-\rho\:)\times\:{x}_{bs})$$

41 $$\:{x}_{average}=\left(\frac{{x}_{1}+{x}_{2}+{x}_{3}}{3}\right)$$

where, $\:\rho\:$ refers to a random number in the range of 0 and 1. $\:{m}_{1}$ and $\:{m}_{2}$ can be assigned using the following equations:

42 $$\:{m}_{1}=\left\{\begin{array}{c}rand\hspace{1em}if\hspace{0.33em}p>0.5\\\:1\hspace{1em}\hspace{1em}\hspace{1em}otherwise\hspace{1em}\end{array}\right.$$

43 $$\:{m}_{2}=\left\{\begin{array}{c}rand\hspace{1em}if\hspace{0.33em}p<0.5\hspace{1em}\hspace{0.33em}\\\:1\hspace{1em}\hspace{1em}\hspace{1em}otherwise\hspace{1em}\end{array}\right.$$

where, $\:\hspace{0.33em}p$ is a random number in the range of [0–1].

## Modified INFO (MINFO)

The MINFO was presented to overcome the shortage of the traditional INFO like the poor diversity between the population, and its stagnation to the local optima. The MINFO is based on two modifications to boost the exploration and the exploitation which can be described as follows:

### Elite centroid quasi-oppositional base learning (ECQOBL)

This modification is implemented to improve the exploitation ability of the proposed optimizer. The Opposition-based learning (OBL) is an efficient common strategy that was employed for accelerating the convergence rate^35^. The OBL is based on assigning the opposite vectors of the current vectors while the quasi-oppositional base learning (QOBL) is based on assigning a vector between the center point and the opposite vector which has been applied for improving the performance of several optimization techniques^36–41^. The OBL and the QOBL can be defined using (44) and (45), respectively.

44 $$\:{x}_{i}^{OBL}={Up}_{i}+{Low}_{i}-{x}_{i}$$

45 $$\:{x}_{i}^{QOBL}=\text{r}\text{a}\text{n}\text{d}\left(\frac{{Up}_{i}+{Low}_{i}}{2},{Up}_{i}+{Low}_{i}-{x}_{i}\right);i=\text{1,2},\dots\:,D$$

The elite average is based on updating the populations based on assigning the average population of the best first three populations. Thus, the populations are ranked based on their values of the objective functions. Then, the average population is determined as described in (46).

46 $$\:{x}_{i}^{CR}=\frac{{{x}_{bs}}_{1}+{{x}_{bs}}_{2}+{{x}_{bs}}_{3}}{3}\:$$

ECQOBL can be expressed as follows:

47 $$\:{x}_{i}^{QOBL}=\text{r}\text{a}\text{n}\text{d}\left(\frac{{Up}_{i}+{Low}_{i}}{2},{Up}_{i}+{Low}_{i}-{x}_{i}^{CR}\right);i=\text{1,2},\dots\:,D$$

Finally, the objective of ECQOBL is to enhance both the convergence speed and accuracy of the proposed algorithms. QOBL works by generating opposite solutions, which accelerates the convergence process, while the Elite Centroid component refines the solutions, thereby improving the overall accuracy of the algorithm.

#### Adaptive levy flight motion

Levy flight motion can be used to increase the searching ability of the populations. ALFM plays a vital role for enhancing the exploration phase via the Lévy flights random walk. Hence, ALFM can improve performance and searching ability of the proposed optimizer. In this strategy the populations will update their locations based on the best solution ($\:{x}_{bs}$), the current solution, levy flight factor ($\:{L}_{F}$) and an adaptive factor ($\:C$) as described in the following equation^42^:

48 $$\:{X}_{new}={rand}_{1}.{x}_{bs}-{rand}_{1}.{X}_{\text{i}}+C\cdot\:{L}_{v}\cdot\:\left({X}_{r}-{X}_{\text{i}}\right)$$

**Fig. 2:**
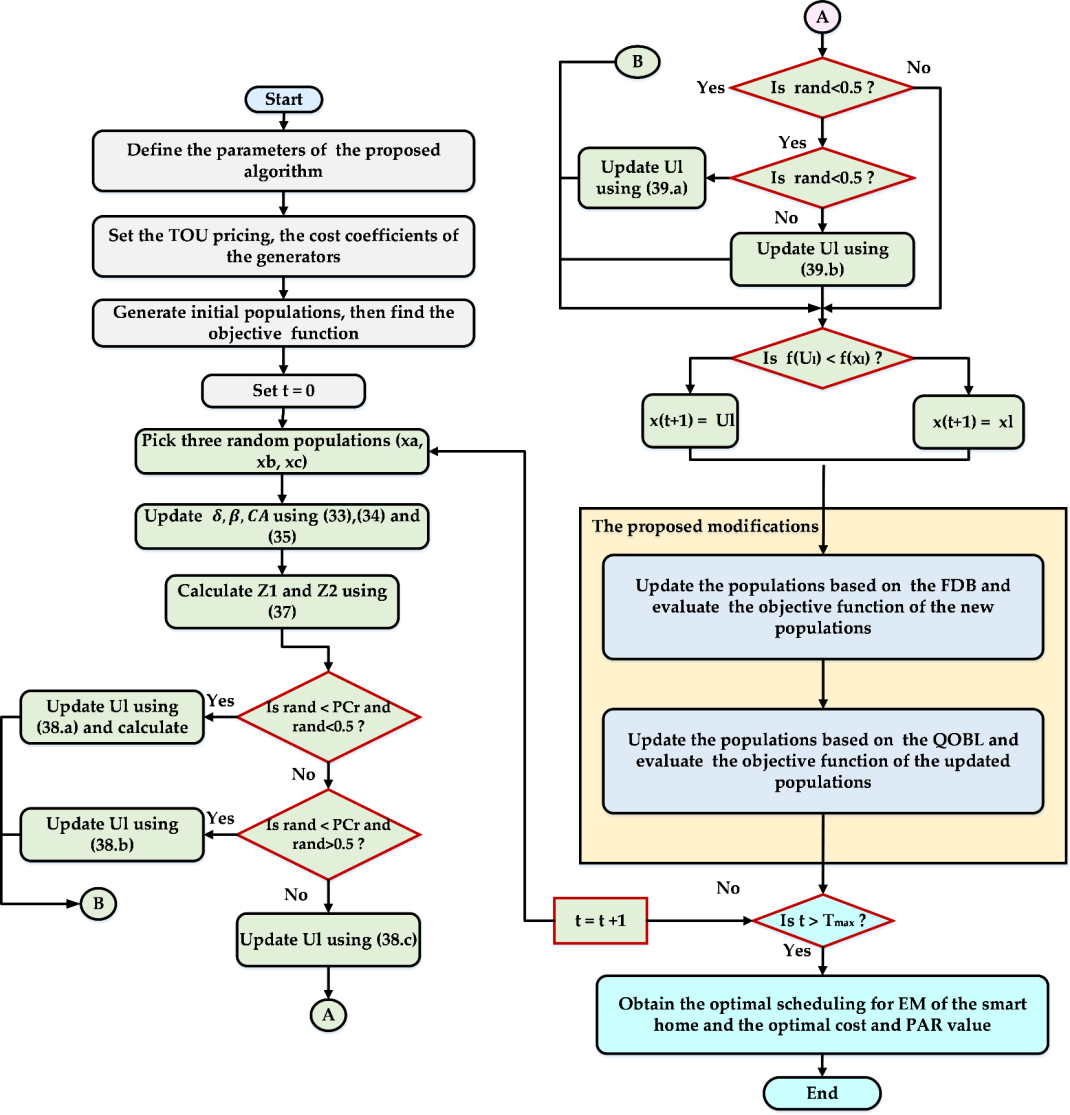
The flow chart of the proposed technique.

In which

49 $$\:C=2\times\:{rand}_{3}\left(1-t/{t}_{\text{m}\text{a}\text{x}}\right)$$

50 $$\:{L}_{v}=0.5\times\:\frac{u\times\:\sigma\:}{|v{|}^{1/{\upphi\:}}}$$

51 $$\:\sigma\:={\left(\frac{{\Gamma\:}(1+\phi\:)\times\:\text{s}\text{i}\text{n}(\pi\:\phi\:/2)}{{\Gamma\:}\left(\right(1+{\upphi\:})/2)\times\:{\upphi\:}\times\:{2}^{({\upphi\:}-1)/2}}\right)}^{1/{\upphi\:}}$$

where $\:{rand}_{1}$and $\:{rand}_{1}$are random factors within rang [0,1]. $\:{X}_{r}$ is a randomly selected vector from the generated vectors in the previous step. $\:v$ and $\:u$ are random values while $\:\phi\:$ is a constant value that equals to 1.5. Figure 2 shows the flow chart of the proposed optimization technique for smart home management.

## Simulation results

The optimal schedule of the smart home is solved by the suggested MINFO. Initially, the performance and efficiency of the proposed MINFO is demonstrated using the CEC-2019 and the basic benchmark functions and the obtained results are compared to other optimization methods including the INFO^19^, sand cat search optimizer (SCSO)^25^, African vultures optimization algorithm (AVOA)^26^, gery wolf optimizer (GWO)^27^, Harris hawks optimization (HHO)^28^, zebra optimization algorithm (ZOA)^29^, whale optimization algorithm (WOA)^30^, RIME algorithm^31^ and Dung beetle optimize (BDO)^32^. It should be highlighted here that the paramters of the intelligent algorithms have been selected to be same as their values in the original puplished papers of each optimizer.

The program code of the MINFO was written using MATLAB 2021a on a core I7 PC that has 2.5 GHz CPU with 32 GB. The numerical results are listed below.

### Application of the MINFO for solving the benchmark functions

#### Description of the benchmark functions

The proposed MINFO is applied to solve the two benchmark functions including 23 basic functions and 10 CEC-2019 functions. The basic functions are categorized in which F1 to F7 are unimodal functions, F8 to F16 and multimodal functions while F17 to F23 are composition functions as described in^43^ while the detail description of CEC-2019 are provided in^44,45^. The parameters of other competitive techniques are tabulated in Table 1 and for fair comparison, number of the search agents and the maximum number of iterations, trial runs are selected the same for all optimizers to be 25, 450 and 20, respectively.

**Table 1:** Parameters of the studied optimization methods.

Algorithm	Parameters
MINFO	$\:c=2,\:d=4,\:\phi\:=1.5$
INFO^19^	$\:c=2,\:d=4$
SCSO^25^	$\:R=\left[-\:2rg,\:2rg\right],\:rg=\left[\text{2,0}\right]\:$
AVOA^26^	$\:L1=0.8$, $\:L2=0.2$, $\:w=2.5$, $\:P1=0.2$, $\:P2=2.5$, $\:P3=2.5$
GWO^27^	$\:a=\left[\text{2,0}\right]$
HHO^28^	$\:\beta\:=1.5$,, $\:{E}_{0}=[-\text{1,1}]$
ZOA^29^	$\:R=0.01$.
WOA^30^	$\:a=\left[\text{2,0}\right]$.
RIME^31^	$\:w=5$.
BDO^32^	$\:k=\lambda\:=1.5,\:b=0.3,\:S=0.5.$

#### The qualitative analysis

**Fig. 3:**
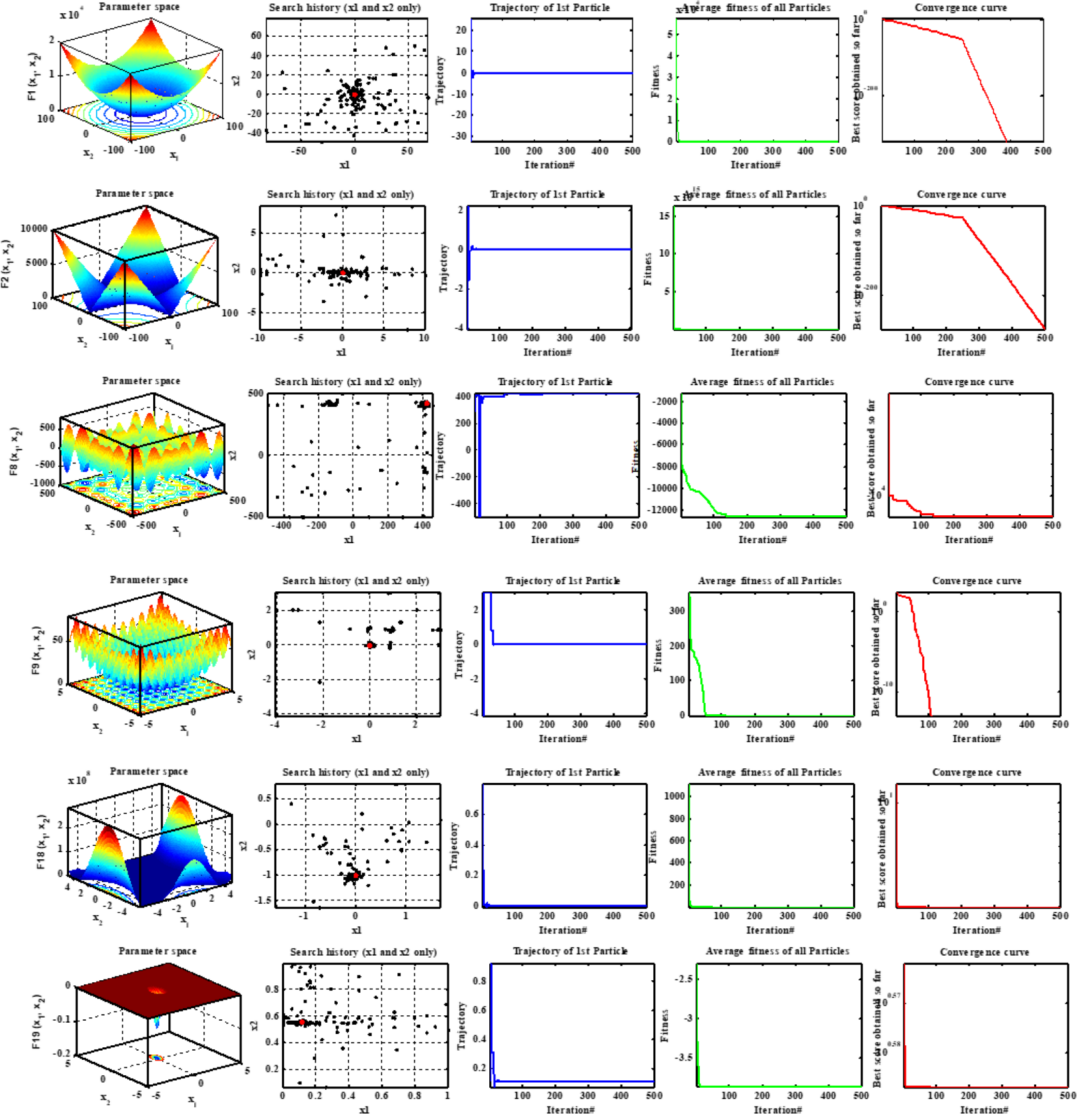
The qualitative results include 3d-view, the searching history, trajectories, the average objective function and the convergence carve.

The qualitative analysis can be used to deduce the robust performance of the proposed algorithm via observing the behavior of the search agents and tends of the objective function during the searching process. Figure 3 shows qualitative results for two unimodal functions (F1-F2), two multimodal functions (F8-F9) and two composition functions (F18-F19). The first column in Fig. 3 is a 3D view of the fitness function which describes the shape and type of the objective function. The second column displays the search history of agents from the first to the last iteration progress which reflects that the proposed MINFO can assign the area in which the fitness function is the low. The third column displays the trend of the trajectory while the fourth and the fifth columns display the average fitness function and convergence carve of the MINFO. According to Fig. 3, the fluctuations of the trajectory at the beginning of the iterative process is high and reduced considerably at the final iteration process which verifies that the proposed MINFO is robust algorithm.

#### Statistical analysis

The statistical outcomes for the proposed MINFO and the INFO, SCSO, AVOA, GWO, HHO, ZOA, WOA, RIME and BDO for the traditional and CEC-2019 are displayed in Tables 2 and 3, respectively. The bolded values refer to the best obtained results. As per the experimental results of Table 2, the proposed MINFO is superior against the other optimization methods for F1 to F8, F14 and F21. Additionally, the performance of the proposed optimizer is identical to other competitive techniques for F9 to F11, and F16 to F20 while its performance is less than other techniques for F12, F13, F15 and F23. Likewise, for the statistical results of Table 3, the suggested MINFO is the best for most functions of the CEC-2019 suite including CEC01, CEC02, CEC05, CEC08, CEC09, and CEC10. Additionally, the performance of the MINFO is the same as the other optimizers for CEC03 while the performance of the MINFO is worse than other techniques for CEC04, CEC6 and CEC7.

#### Convergence analysis

**Fig. 4:**
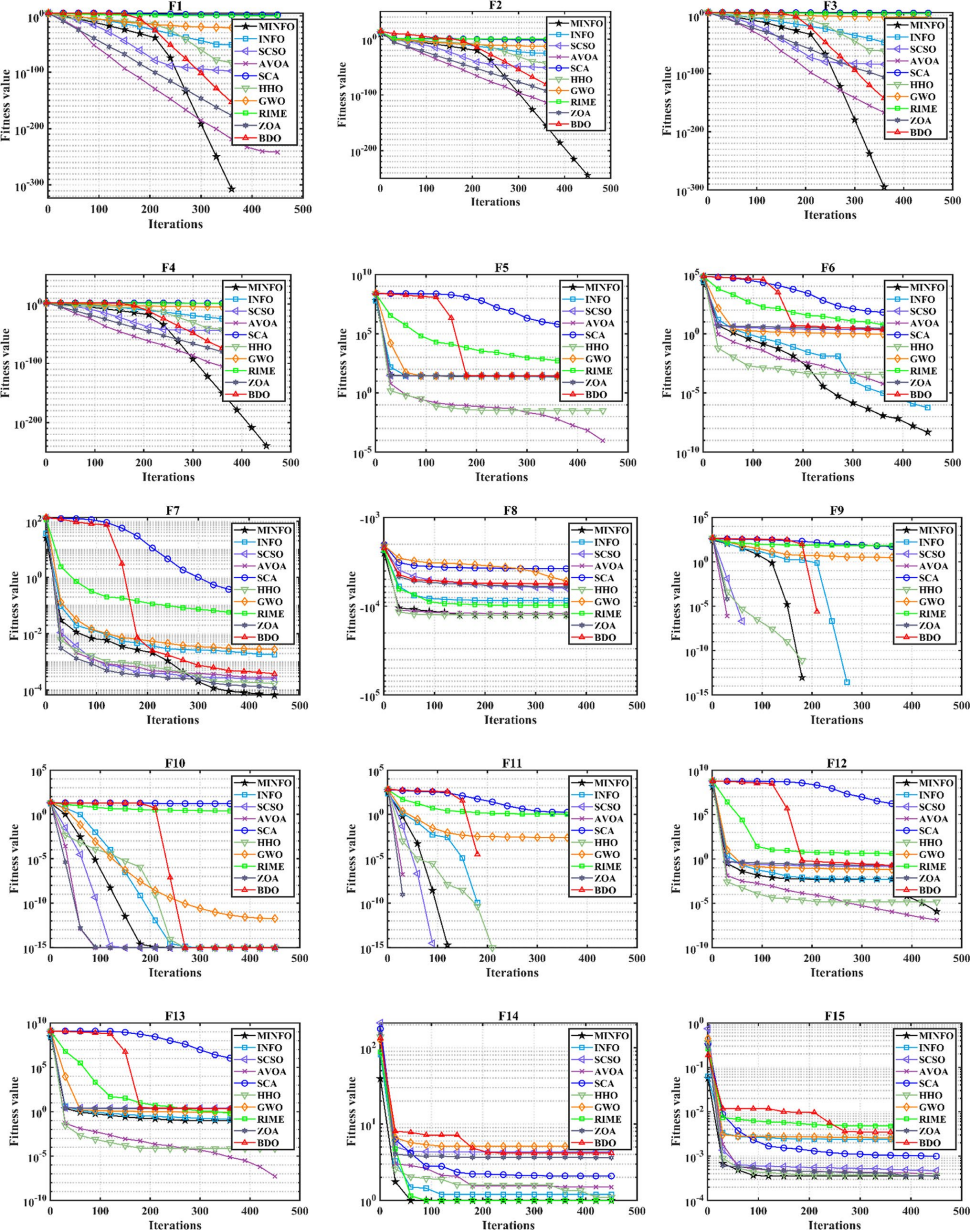

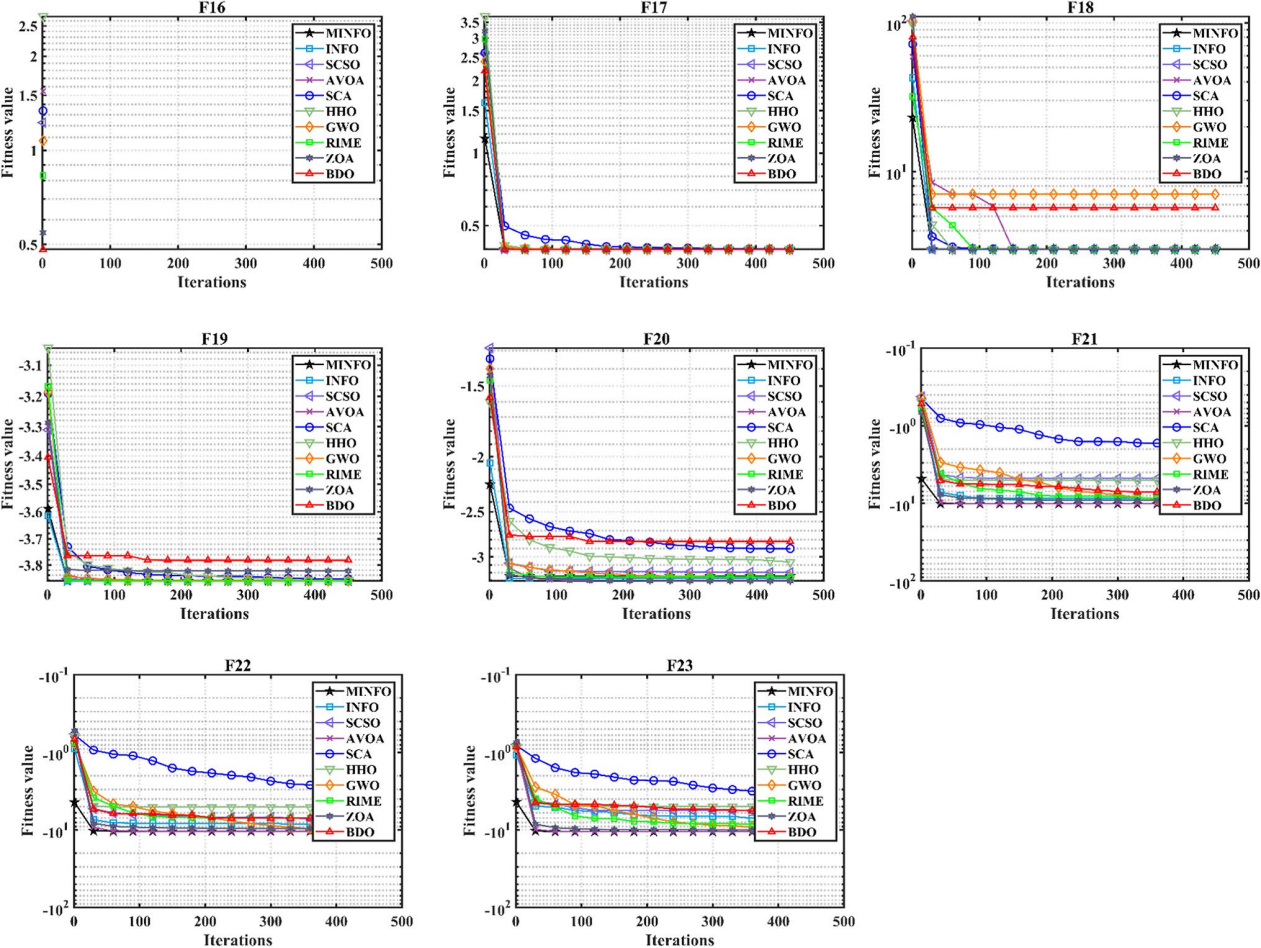
The convergence curves of the MINFO and the other optimization methods for the traditional benchmark functions.

**Fig. 5:**
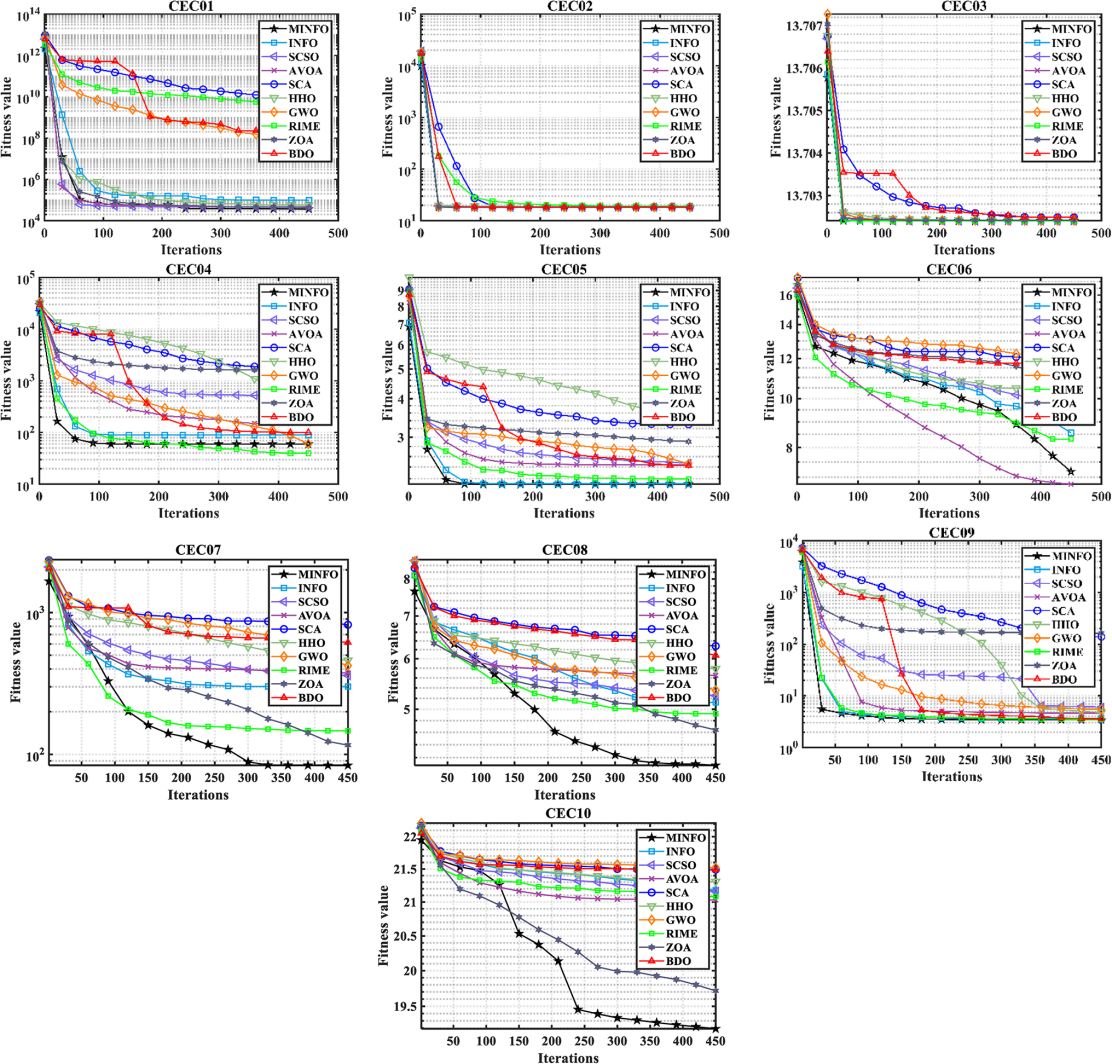
The convergence curves of the MOINFO and the other optimization methods for the CEC-2019 test.

The trends of the traditional and CEC-2019 test functions are displayed in Figs. 4 and 5, respectively. According to Figs. 4 and 5, the convergence characteristics of proposed MINFO are the best for most of the test functions where it converged rapidly compared to the INFO, SCSO, AVOA, GWO, HHO, ZOA, WOA, RIME and BDO. However, there are some optimizers that have convergence characteristic better than the suggested MINFO like F5, F12 and F13, the AVOA and HHO are the best while PROME is the best for CEC04 and AVOA is the best for CEC6.

#### Boxplot analysis

**Fig. 6:**
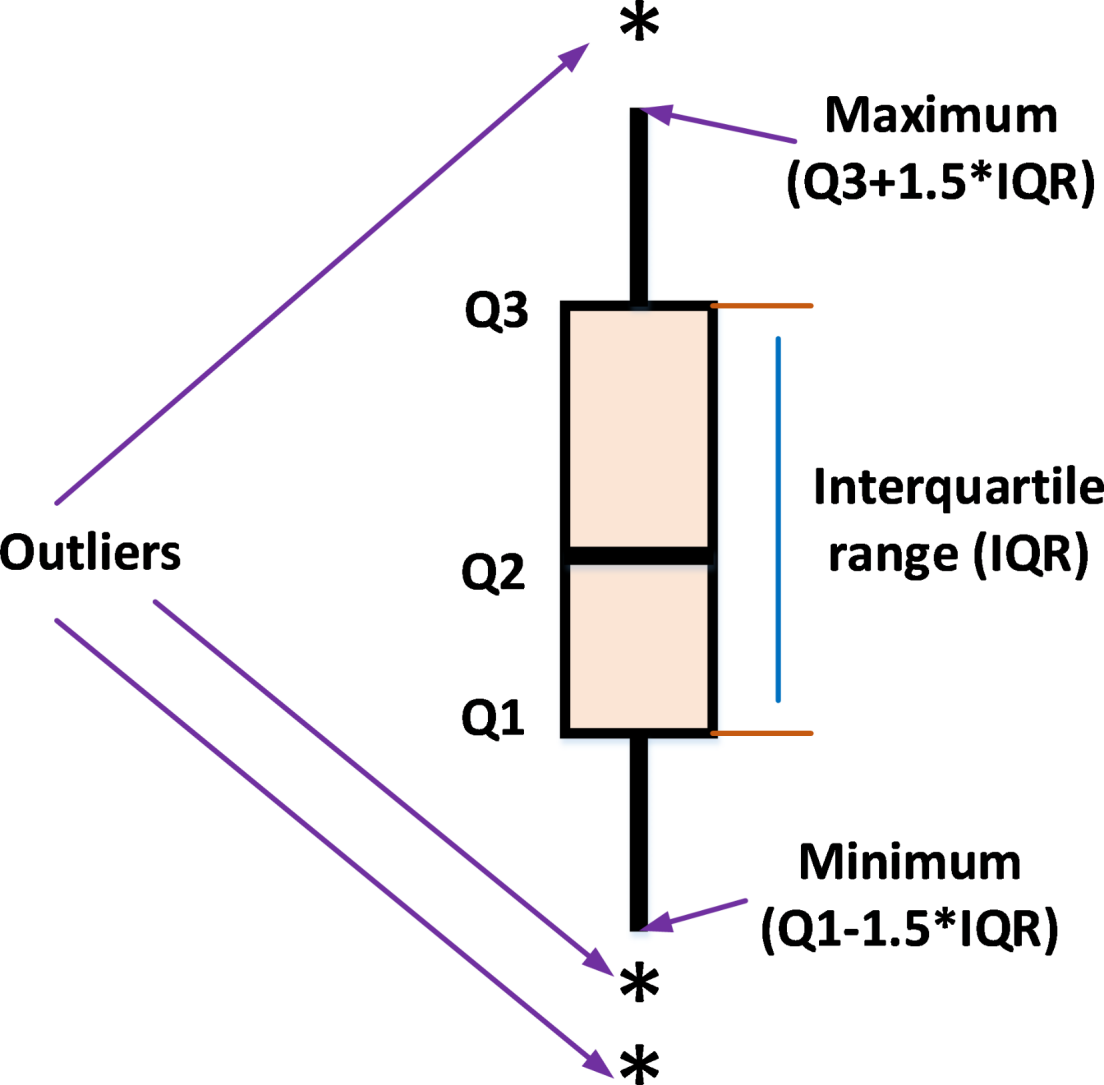
The boxplot representation.

**Fig. 7:**
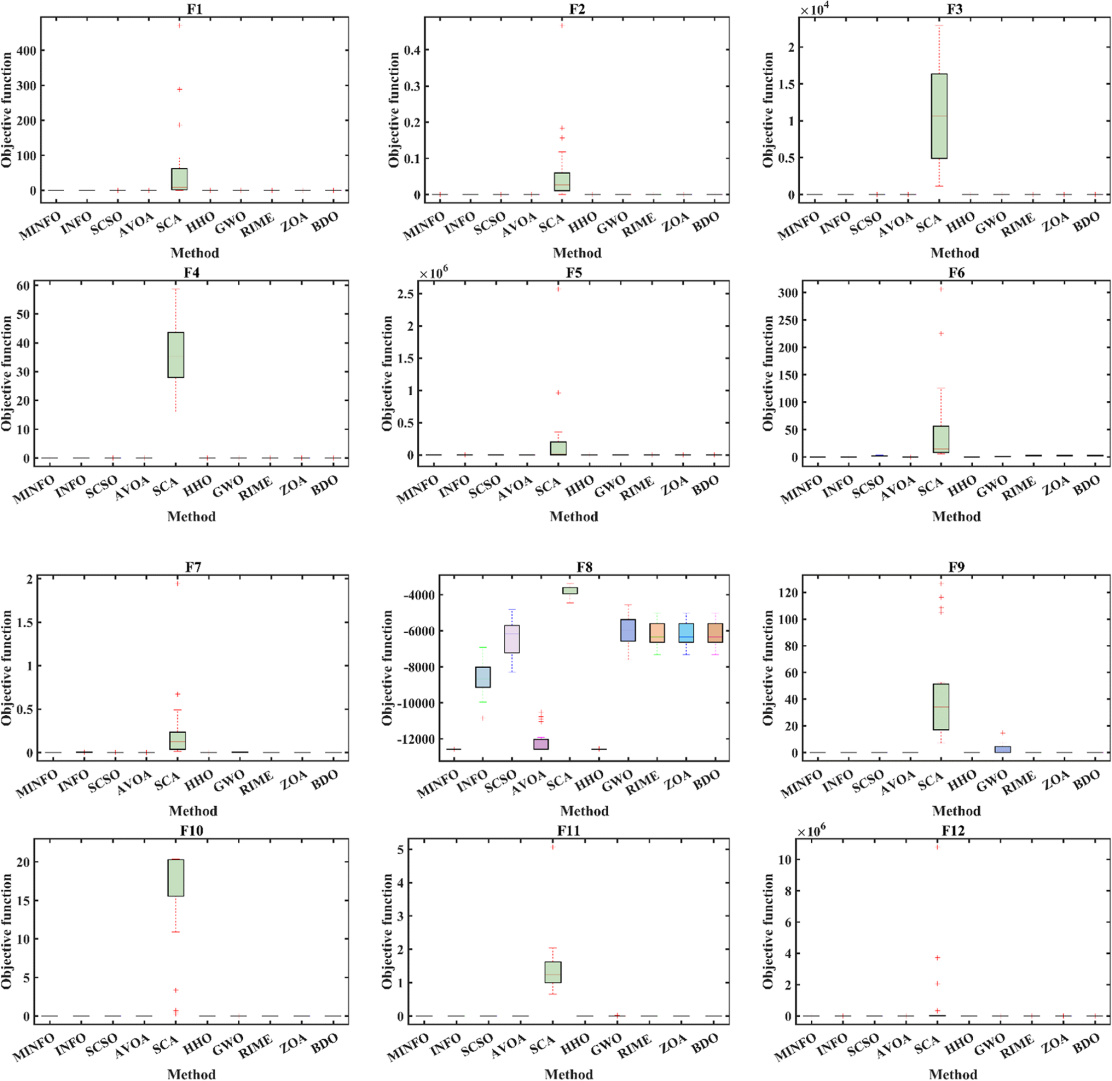

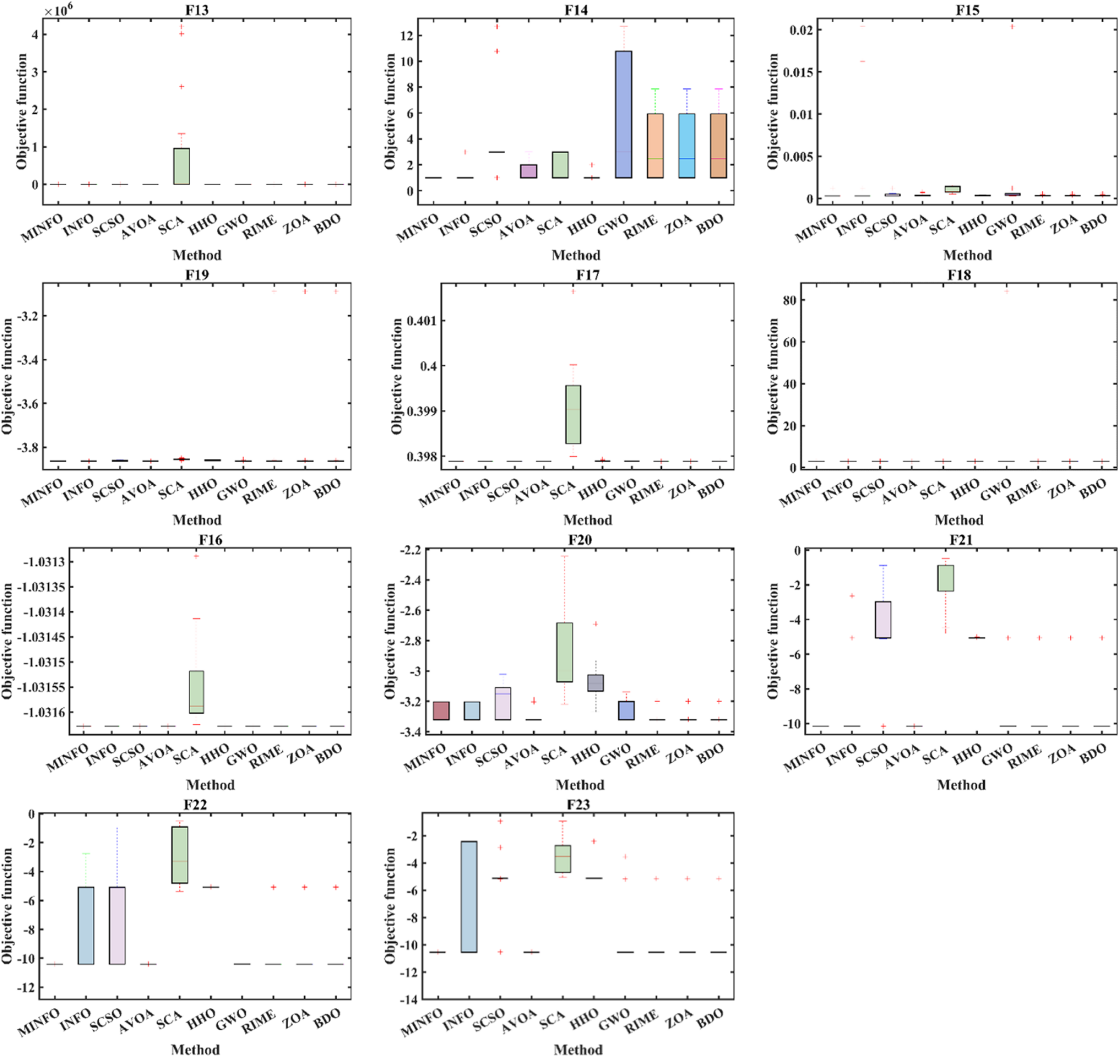
The convergence plots for the traditional benchmark functions.

**Fig. 8:**
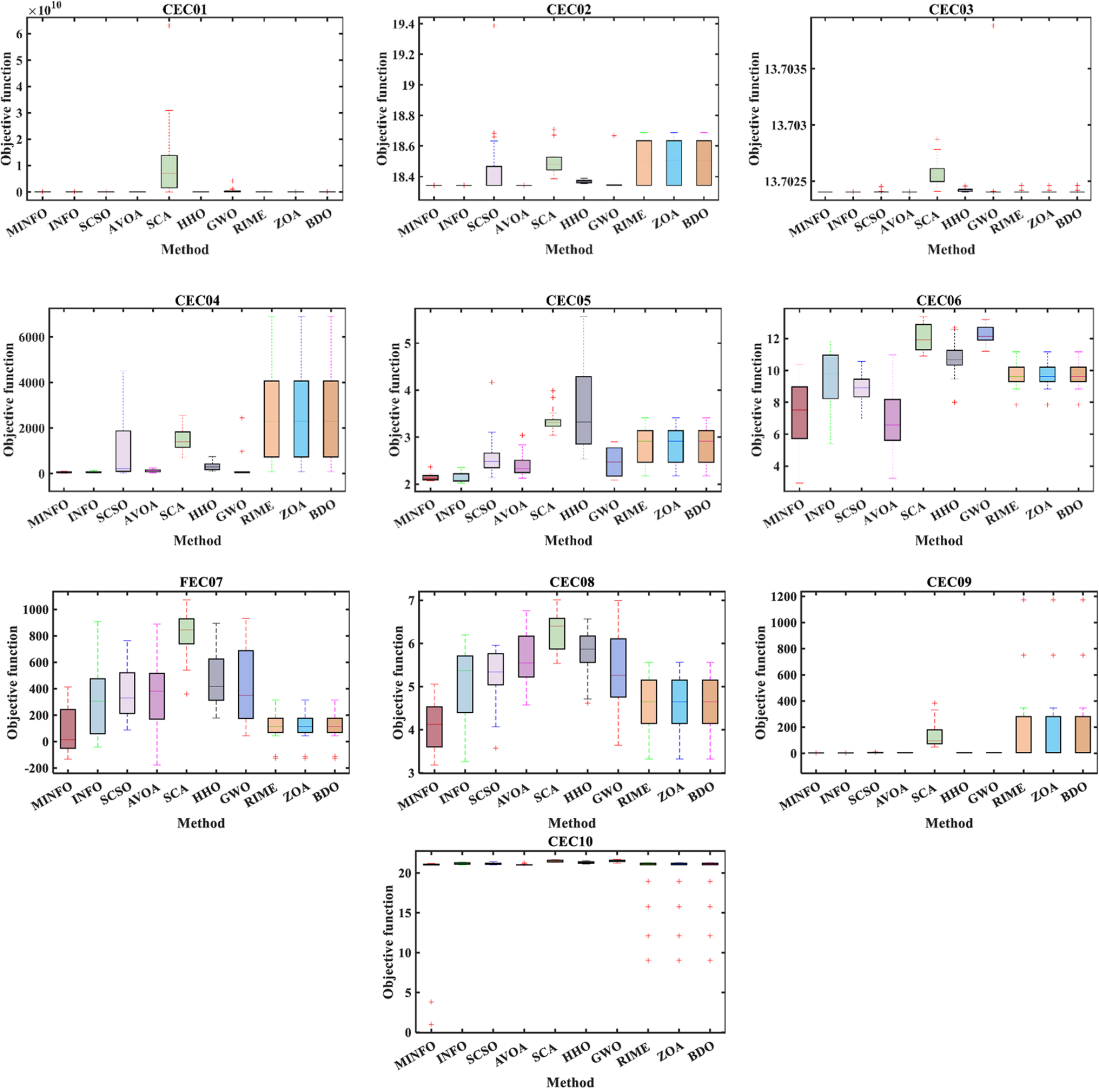
The convergence plots for the CEC-2019 functions.

boxplot is an efficient way to display the distribution of the obtained numerical results. As shown in Fig. 6, the inside line of the boxplot is denoted Q2 which represents the median value while Q1 and Q3 are the 25th and the 75th percentiles, respectively. The points that have values more than the maximum or less than the minimum known at outliers. Figures 7 and 8 display the boxplot for the two test suites. Judging from Figs. 7 and 8, the distribution of the data for the most function where it has the narrowest boxplot and the minimum median value.

**Table 2:** The statistical results of the competitive techniques for the basic test suite.

Function	Technique	Mean	Best	Average	SD
F1	MINFO	0	0	0	0
	INFO	2.516E-53	4.134E-54	5.453E-53	1.48E-53
	SCSO	2.34E-100	3.012E-110	3.981E-99	8.881E-100
	AVOA	1.295E-242	1.66E-302	2.191E-241	0.000
	SCA	3.725E + 01	3.801E-02	2.125E + 02	5.474E + 01
	HHO	2.773E-84	2.136E-99	4.477E-83	1.04E-83
	GWO	1.323E-22	4.569E-24	7.667E-22	1.84E-22
	RIME	2.667	1.426	6.491	1.285
	ZOA	1.994E-222	7.34E-234	3.984E-221	0
	DBO	9.365E-164	1.319E-179	1.842E-162	0
F2	MINFO	5.741E-245	3.282E-253	4.529E-244	0
	INFO	2.23E-26	9.660E-27	3.880E-26	7.744E-27
	SCSO	9.255E-53	1.597E-58	1.761E-51	3.929E-52
	AVOA	4.968E-123	1.211E-156	9.936E-122	2.222E-122
	SCA	6.459E-02	2.422E-4	4.671E-01	1.78E-01
	HHO	1.619E-44	5.563E-54	2.788E-43	6.226E-44
	GWO	5.654E-14	1.37E-14	1.344E-13	3.639E-14
	RIME	1.481	8.028E-01	4.601	9.219E-01
	ZOA	3.998E-117	3.700E-122	4.650E-116	1.177E-116
	DBO	4.766E-88	1.67E-91	7.571E-87	1.682E-87
F3	MINFO	0	0	0	0
	INFO	2.91E-50	4.121E-52	6.698E-50	1.956E-50
	SCSO	8.619E-86	7.841E-97	1.65E-84	3.578E-85
	AVOA	3.259E-185	1.686E-245	6.517E-184	0.000
	SCA	1.89E + 4	1.138E + 3	2.290E + 4	6.724E + 3
	HHO	8.465E-62	1.570E-84	9.342E-61	2.421E-61
	GWO	4.918E-4	7.694E-7	2.249E-3	6.62E-4
	RIME	1.746E + 3	8.488E + 02	3.317E + 3	6.295E + 02
	ZOA	3.418E-138	7.523E-154	6.436E-137	1.437E-137
	DBO	2.813E-148	2.30E-166	2.911E-147	8.659E-148
F4	MINFO	4.816E-240	4.687E-249	6.319E-239	0
	INFO	3.511E-27	9.398E-28	6.986E-27	1.587E-27
	SCSO	3.518E-45	2.212E-50	4.769E-44	1.14E-44
	AVOA	4.715E-117	6.881E-147	9.431E-116	2.19E-116
	SCA	3.659E + 01	1.614E + 01	5.866E + 01	1.170E + 01
	HHO	1.282E-43	1.117E-50	2.218E-42	4.943E-43
	GWO	1.677E-5	2.125E-6	5.559E-5	1.372E-5
	RIME	1.72E + 01	4.417	1.952E + 01	4.246
	ZOA	1.950E-102	3.222E-16	2.84E-101	6.241E-102
	DBO	7.147E-79	6.277E-88	8.535E-78	2.63E-78
F5	MINFO	2.282E + 01	2.186E + 01	2.396E + 01	5.829E-01
	INFO	2.387E + 01	2.316E + 01	2.542E + 01	5.659E-01
	SCSO	2.819E + 01	2.621E + 01	2.887E + 01	8.151E-01
	AVOA	9.377E-5	4.64E-6	2.629E-4	7.958E-5
	SCA	2.450E + 5	1.430E + 02	2.570E + 6	5.99E + 5
	HHO	3.219E-02	1.330E-3	1.173E-01	3.472E-02
	GWO	2.746E + 01	2.611E + 01	2.877E + 01	9.021E-01
	RIME	3.980E + 02	8.621E + 01	2.489E + 3	5.231E + 02
	ZOA	2.856E + 01	2.783E + 01	2.885E + 01	3.161E-01
	DBO	2.712E + 01	2.597E + 01	2.876E + 01	8.349E-01
F6	MINFO	4.785E-9	1.328E-10	1.496E-8	4.649E-9
	INFO	5.782E-7	3.518E-9	6.157E-6	1.370E-6
	SCSO	2.147	1.269	3.480	5.252E-01
	AVOA	3.698E-6	9.678E-8	3.666E-5	7.963E-6
	SCA	5.330E + 01	5.727	3.62E + 02	8.40E + 01
	HHO	3.822E-4	5.61E-6	1.523E-3	4.995E-4
	GWO	9.015E-01	2.511E-01	1.764	4.020E-01
	RIME	2.865	1.527	6.476	1.130
	ZOA	2.920	1.85	4.131	7.800E-01
	DBO	9.415E-01	3.845E-01	1.723	3.616E-01
F7	MINFO	6.597E-5	3.760E-6	1.715E-4	4.844E-5
	INFO	1.799E-3	3.834E-4	4.792E-3	1.133E-3
	SCSO	2.454E-4	3.332E-7	1.47E-3	3.96E-4
	AVOA	2.784E-4	1.58E-7	9.325E-4	2.393E-4
	SCA	2.622E-01	1.233E-02	1.944	4.345E-01
	HHO	1.683E-4	1.935E-6	1.184E-3	2.68E-4
	GWO	2.769E-3	5.470E-4	5.162E-3	1.355E-3
	RIME	5.30E-02	1.989E-02	9.443E-02	1.997E-02
	ZOA	1.154E-4	6.545E-6	3.98E-4	8.24E-5
	DBO	3.739E-4	7.667E-5	1.360E-3	3.471E-4
F8	MINFO	-1.257E + 4	-1.257E + 4	-1.257E + 4	5.252E-4
	INFO	-8.643E + 3	-1.85E + 4	-6.922E + 3	9.250E + 02
	SCSO	-6.438E + 3	-8.290E + 3	-4.813E + 3	9.716E + 02
	AVOA	-1.216E + 4	-1.257E + 4	-1.52E + 4	7.29E + 02
	SCA	-3.86E + 3	-4.446E + 3	-3.384E + 3	2.669E + 02
	HHO	-1.257E + 4	-1.257E + 4	-1.256E + 4	2.888
	GWO	-6.08E + 3	-7.611E + 3	-4.566E + 3	8.167E + 02
	RIME	-9.788E + 3	-1.91E + 4	-8.759E + 3	5.538E + 02
	ZOA	-6.219E + 3	-7.334E + 3	-5.42E + 3	6.783E + 02
	DBO	-5.559E + 3	-8.397E + 3	-3.366E + 3	1.394E + 3
F9	MINFO	0	0	0	0
	INFO	0	0	0	0
	SCSO	0	0	0	0
	AVOA	0	0	0	0
	SCA	4.501E + 01	7.66	1.266E + 02	3.827E + 01
	HHO	0.000	0.000	0.000	0.000
	GWO	2.787	1.137E-13	1.467E + 01	3.660
	RIME	6.554E + 01	3.780E + 01	9.354E + 01	1.431E + 01
	ZOA	0	0	0	0
	DBO	0	0	0	0
F10	MINFO	8.882E-16	8.882E-16	8.882E-16	0
	INFO	8.882E-16	8.882E-16	8.882E-16	0
	SCSO	8.882E-16	8.882E-16	8.882E-16	0
	AVOA	8.882E-16	8.882E-16	8.882E-16	0
	SCA	1.598E + 01	2.612E-01	2.34E + 01	7.889
	HHO	8.882E-16	8.882E-16	8.882E-16	0.000
	GWO	1.785E-12	4.556E-13	4.30E-12	8.984E-13
	RIME	2.325	1.217	3.92	5.36E-01
	ZOA	8.882E-16	8.882E-16	8.882E-16	0
	DBO	8.882E-16	8.882E-16	8.882E-16	0
F11	MINFO	0	0	0	0
	INFO	0	0	0	0
	SCSO	0	0	0	0
	AVOA	0	0	0	0
	SCA	1.475	6.600E-01	5.62	9.243E-01
	HHO	0	0	0	0
	GWO	2.236E-3	0.000	1.901E-02	5.583E-3
	RIME	1.07	9.238E-01	1.46	3.227E-02
	ZOA	0.000	0.000	0.000	0.000
	DBO	0	0	0	0
F12	MINFO	1.211E-6	1.597E-11	2.421E-5	5.413E-6
	INFO	5.183E-3	1.244E-10	1.37E-01	2.318E-02
	SCSO	1.268E-01	5.555E-02	2.337E-01	5.915E-02
	AVOA	1.328E-7	5.671E-9	9.17E-7	1.902E-7
	SCA	8.466E + 5	2.598	1.78E + 7	2.515E + 6
	HHO	1.453E-5	2.601E-9	4.973E-5	1.698E-5
	GWO	5.690E-02	1.279E-02	1.46E-01	4.016E-02
	RIME	3.935	2.878E-01	1.356E + 01	3.42
	ZOA	1.818E-01	5.741E-02	4.246E-01	9.145E-02
	DBO	3.845E-02	7.846E-3	8.298E-02	1.897E-02
F13	MINFO	1.39E-01	4.654E-10	4.165E-01	1.161E-01
	INFO	1.42E-01	2.264E-7	4.65E-01	1.07E-01
	SCSO	2.545	1.591	2.891	2.749E-01
	AVOA	5.315E-8	4.930E-9	1.138E-7	3.935E-8
	SCA	7.134E + 5	6.67	4.24E + 6	1.340E + 6
	HHO	7.131E-5	3.212E-6	2.08E-4	5.531E-5
	GWO	7.593E-01	2.443E-01	1.188	2.458E-01
	RIME	3.938E-01	1.935E-01	8.900E-01	1.642E-01
	ZOA	2.299	1.393	2.878	3.469E-01
	DBO	1.710	4.624E-01	2.974	8.945E-01
F14	MINFO	9.980E-01	9.980E-01	9.980E-01	0
	INFO	1.196	9.980E-01	2.982	6.17E-01
	SCSO	4.336	9.980E-01	1.267E + 01	3.632
	AVOA	1.494	9.980E-01	2.982	8.27E-01
	SCA	2.90	9.980E-01	2.982	1.012
	HHO	1.97	9.980E-01	1.992	3.60E-01
	GWO	5.15	9.980E-01	1.267E + 01	4.636
	RIME	9.980E-01	9.980E-01	9.980E-01	7.687E-12
	ZOA	3.611	9.980E-01	7.874	2.559
	DBO	4.181	9.980E-01	1.267E + 01	4.127
F15	MINFO	3.533E-4	3.75E-4	1.223E-3	2.48E-4
	INFO	2.201E-3	3.75E-4	2.36E-02	5.560E-3
	SCSO	4.695E-4	3.75E-4	1.223E-3	2.778E-4
	AVOA	4.16E-4	3.75E-4	7.651E-4	1.619E-4
	SCA	9.971E-4	5.151E-4	1.583E-3	3.626E-4
	HHO	3.700E-4	3.95E-4	4.345E-4	3.734E-5
	GWO	2.502E-3	3.228E-4	2.36E-02	6.114E-3
	RIME	4.745E-3	5.877E-4	2.36E-02	8.016E-3
	ZOA	3.516E-4	3.75E-4	5.694E-4	7.29E-5
	DBO	3.282E-3	3.75E-4	5.654E-02	1.254E-02
F16	MINFO	-1.32	-1.32	-1.32	1.021E-12
	INFO	-1.32	-1.32	-1.32	1.96E-16
	SCSO	-1.32	-1.32	-1.32	1.131E-9
	AVOA	-1.32	-1.32	-1.32	1.441E-16
	SCA	-1.32	-1.32	-1.32	8.153E-5
	HHO	-1.32	-1.32	-1.32	2.134E-9
	GWO	-1.32	-1.32	-1.32	4.140E-8
	RIME	-1.32	-1.32	-1.32	3.533E-7
	ZOA	-1.32	-1.32	-1.32	3.490E-9
	DBO	-1.32	-1.32	-1.32	1.248E-16
F17	MINFO	3.979E-01	3.979E-01	3.979E-01	0.000
	INFO	3.979E-01	3.979E-01	3.979E-01	0.000
	SCSO	3.979E-01	3.979E-01	3.979E-01	2.847E-8
	AVOA	3.979E-01	3.979E-01	3.979E-01	3.979E-16
	SCA	3.991E-01	3.980E-01	4.016E-01	8.882E-4
	HHO	3.979E-01	3.979E-01	3.979E-01	1.424E-5
	GWO	3.979E-01	3.979E-01	3.979E-01	9.692E-7
	RIME	3.979E-01	3.979E-01	3.979E-01	4.52E-8
	ZOA	3.979E-01	3.979E-01	3.979E-01	1.370E-8
	DBO	3.979E-01	3.979E-01	3.979E-01	0.000
F18	MINFO	3	3	3	3.222E-16
	INFO	3	3	3	6.910E-16
	SCSO	3	3	3	1.84E-5
	AVOA	3	3	3	6.940E-6
	SCA	3	3	3	3.131E-4
	HHO	3	3	3	2.938E-6
	GWO	3	3	3	1.811E + 01
	RIME	3	3	3	6.522E-7
	ZOA	3	3	3	2.944E-5
	DBO	5.700	3.000	3.000E + 01	8.310
F19	MINFO	-3.86278	-3.86278	-3.86278	2.28E-15
	INFO	-3.86278	-3.86278	-3.86278	2.26E-15
	SCSO	-3.8683	-3.86278	-3.8549	0.03296
	AVOA	-3.86278	-3.86278	-3.86278	9.3E-12
	SCA	-3.8545	-3.8685	-3.8485	0.0357
	HHO	-3.85986	-3.86269	-3.8559	0.002399
	GWO	-3.8624	-3.86278	-3.8549	0.001816
	RIME	-3.86278	-3.86278	-3.86278	1.21E-6
	ZOA	-3.82357	-3.86278	-3.8976	0.172721
	DBO	-3.78154	-3.86278	-3.8976	0.236613
F20	MINFO	-3.24471	-3.322	-3.231	0.58182
	INFO	-3.27444	-3.322	-3.231	0.59759
	SCSO	-3.19326	-3.32199	-3.0268	0.1538
	AVOA	-3.3345	-3.322	-3.18894	0.45364
	SCA	-2.9094	-3.21769	-2.24529	0.24677
	HHO	-3.6449	-3.26985	-2.68972	0.120173
	GWO	-3.25857	-3.32198	-3.13743	0.74741
	RIME	-3.25656	-3.32198	-3.234	0.667
	ZOA	-3.3953	-3.32199	-3.19891	0.3767
	DBO	-2.82012	-3.322	-1.26768	0.663247
F21	MINFO	-10.1532	-10.1532	-10.1532	3.5E-15
	INFO	-8.8984	-10.1532	-2.6347	2.648644
	SCSO	-4.77873	-10.153	-0.8898	2.933737
	AVOA	-10.1532	-10.1532	-10.1532	3.68E-12
	SCA	-1.72763	-4.67001	-0.49652	1.357247
	HHO	-5.4882	-5.5515	-5.0622	0.01549
	GWO	-9.64179	-10.1531	-5.5518	1.568585
	RIME	-8.5198	-10.1532	-2.6341	2.618871
	ZOA	-9.13351	-10.1532	-5.5519	2.92133
	DBO	-7.61787	-10.1532	-5.552	2.601243
F22	MINFO	-10.4029	-10.4029	-10.4029	3.58E-15
	INFO	-8.43985	-10.4029	-2.7659	3.12288
	SCSO	-7.15577	-10.4029	-0.988	3.175932
	AVOA	-10.4029	-10.4029	-10.4029	6.5E-12
	SCA	-2.98854	-5.36298	-0.52389	1.83283
	HHO	-5.8255	-5.8767	-5.585	0.06533
	GWO	-10.401	-10.4027	-10.3982	0.001363
	RIME	-6.9576	-10.4028	-2.75181	3.38739
	ZOA	-9.6554	-10.4029	-5.8752	1.947211
	DBO	-7.31424	-10.4029	-1.83759	3.255521
F23	MINFO	-10.5364	-10.5364	-10.5364	3.36E-15
	INFO	-9.0368	-10.5364	-2.42173	3.15622
	SCSO	-6.29279	-10.5364	-0.94888	3.65496
	AVOA	-10.5364	-10.5364	-10.5364	3.54E-12
	SCA	-3.51668	-6.0885	-0.55412	1.639184
	HHO	-5.36469	-10.0238	-5.9712	1.96674
	GWO	-9.72275	-10.5362	-5.12843	1.980133
	RIME	-8.01561	-10.5362	-2.4273	3.259847
	ZOA	-9.18437	-10.5364	-5.12832	2.402523
	DBO	-6.83165	-10.5364	-1.85948	3.575711

**Table 3:** The statistical results of the competitive techniques for the cec-2019 test suite.

Function	Technique	Mean	Best	Average	SD
CEC01	MINFO	37255.34	34162.17	3843.76	1194.647
	INFO	10372.8	36537.74	54992.3	13328.9
	SCSO	45424.71	39756.42	53702.55	3516.887
	AVOA	46325.72	41797.6	54628.87	3493.645
	SCA	1.02E + 10	1.28E + 8	5.45E + 10	1.38E + 10
	HHO	55745.89	45369.95	8730.11	8793.633
	GWO	90,150,154	625880.6	3.47E + 8	1.11E + 8
	RIME	4.94E + 9	8.27E + 8	1.45E + 10	3.49E + 9
	ZOA	43989.18	38511.2	55330.12	4265.428
	DBO	1.55E + 8	37980.57	3.01E + 9	6.72E + 8
CEC02	MINFO	18.34286	18.34286	18.34286	3.65E-15
	INFO	18.34286	18.34286	18.34286	3.46E-15
	SCSO	18.3955	18.34298	18.7153	0.12718
	AVOA	18.34289	18.34286	18.34344	0.000131
	SCA	18.5472	18.39573	19.22287	0.174951
	HHO	18.3629	18.34469	18.3947	0.09782
	GWO	18.37784	18.34351	18.68246	0.13885
	RIME	18.95334	18.51333	20.2915	0.436781
	ZOA	18.49484	18.34294	19.1779	0.299911
	DBO	18.34286	18.34286	18.34286	7.99E-15
CEC03	MINFO	13.7024	13.7024	13.7024	1.82E-15
	INFO	13.7024	13.7024	13.7024	2.8E-15
	SCSO	13.7024	13.7024	13.70242	2.82E-6
	AVOA	13.7024	13.7024	13.7024	1.5E-10
	SCA	13.70249	13.70241	13.70267	8.37E-5
	HHO	13.70241	13.70241	13.70244	8.7E-6
	GWO	13.70241	13.7024	13.70245	9.36E-6
	RIME	13.7024	13.7024	13.7024	3.8E-9
	ZOA	13.70242	13.7024	13.70246	2.4E-5
	DBO	13.70249	13.7024	13.7393	0.0034
CEC04	MINFO	59.65223	18.9923	118.435	32.72963
	INFO	89.30131	21.89412	246.7478	52.7984
	SCSO	500.7863	41.48317	2626.76	755.263
	AVOA	148.887	49.75314	339.2481	67.4255
	SCA	1586.115	792.3018	388.771	687.846
	HHO	300.813	82.50261	917.1854	197.641
	GWO	60.1903	36.56375	114.2436	17.1732
	RIME	39.75241	21.77548	63.34396	11.51874
	ZOA	1610.42	92.4541	359.498	179.122
	DBO	100.3348	20.9886	492.3433	114.9192
CEC05	MINFO	2.112325	2.02737	2.292796	0.6229
	INFO	2.120171	2.41797	2.351767	0.72859
	SCSO	2.47794	2.133979	3.276132	0.282897
	AVOA	2.444168	2.58974	3.24343	0.3756
	SCA	3.3783	3.03949	3.84438	0.1863
	HHO	3.512317	2.49645	4.647325	0.592774
	GWO	2.461347	2.96002	2.8496	0.24264
	RIME	2.195786	2.45471	2.489925	0.117959
	ZOA	2.98168	2.24336	4.23814	0.56256
	DBO	2.432291	2.78969	3.23975	0.382871
CEC06	MINFO	7.17398	4.292326	10.2385	1.85923
	INFO	8.55598	3.869728	11.6446	2.256713
	SCSO	9.15114	5.96236	11.62123	1.602949
	AVOA	6.77641	2.96346	10.81534	2.195123
	SCA	12.3387	10.17901	13.02579	0.767986
	HHO	10.3479	7.532948	12.55518	1.245523
	GWO	12.16178	10.87874	13.23839	0.661536
	RIME	8.321724	5.897	11.13728	1.367531
	ZOA	9.420137	6.96288	10.94481	0.871562
	DBO	11.67967	9.436441	13.67237	1.180133
CEC07	MINFO	85.33118	-100.634	320.9922	118.7166
	INFO	301.7563	-282.85	596.6996	238.8736
	SCSO	421.3142	171.3227	963.797	28.8451
	AVOA	339.3975	-16.659	937.469	257.8348
	SCA	845.6922	425.7591	1212.498	252.2629
	HHO	428.5881	68.9866	750.581	151.5161
	GWO	386.363	84.6625	875.858	237.5194
	RIME	144.5547	-15.968	410.4766	166.739
	ZOA	133.2917	-123.874	302.2567	122.4622
	DBO	753.9699	273.3554	1236.476	287.2417
CEC08	MINFO	4.001148	2.333595	5.652865	0.894526
	INFO	4.877421	3.625484	6.326711	0.75361
	SCSO	5.43954	4.525624	6.574659	0.62457
	AVOA	5.733117	4.396865	7.161853	0.69354
	SCA	6.228756	5.52539	6.734893	0.344389
	HHO	5.88374	4.968978	7.269386	0.575978
	GWO	5.191392	3.25011	7.251018	0.933636
	RIME	4.97928	3.510126	5.81533	0.647837
	ZOA	4.85944	4.37255	5.602875	0.340027
	DBO	6.255297	5.211678	6.940025	0.49179
CEC09	MINFO	3.445651	3.348834	3.98798	0.137517
	INFO	3.622533	3.399488	4.54487	0.277645
	SCSO	37.9657	4.198365	357.658	98.88756
	AVOA	4.43635	3.575364	5.584447	0.641955
	SCA	155.7332	29.74674	402.9924	101.3556
	HHO	4.592322	3.765138	6.61797	0.613755
	GWO	6.3327	4.168	7.984785	1.06288
	RIME	3.56026	3.441022	3.814597	0.100252
	ZOA	56.6698	4.932742	388.2395	119.828
	DBO	3.66584	3.373155	4.28655	0.263178
CEC10	MINFO	20.2363	5.029776	21.16952	3.57962
	INFO	21.1606	21.00168	21.42476	0.146331
	SCSO	21.2869	21.01293	21.41739	0.114925
	AVOA	21.0284	20.995	21.17879	0.56648
	SCA	21.4942	21.27799	21.64584	0.95178
	HHO	21.33021	21.9838	21.64415	0.160164
	GWO	20.87852	8.29326	21.64768	2.96313
	RIME	21.1767	21.3959	21.22451	0.5732
	ZOA	20.8193	14.72452	21.27269	1.435731
	DBO	21.44671	21.0566	21.65456	0.177656

#### Wilcoxon rank sum and Friedman tests

**Fig. 9:**
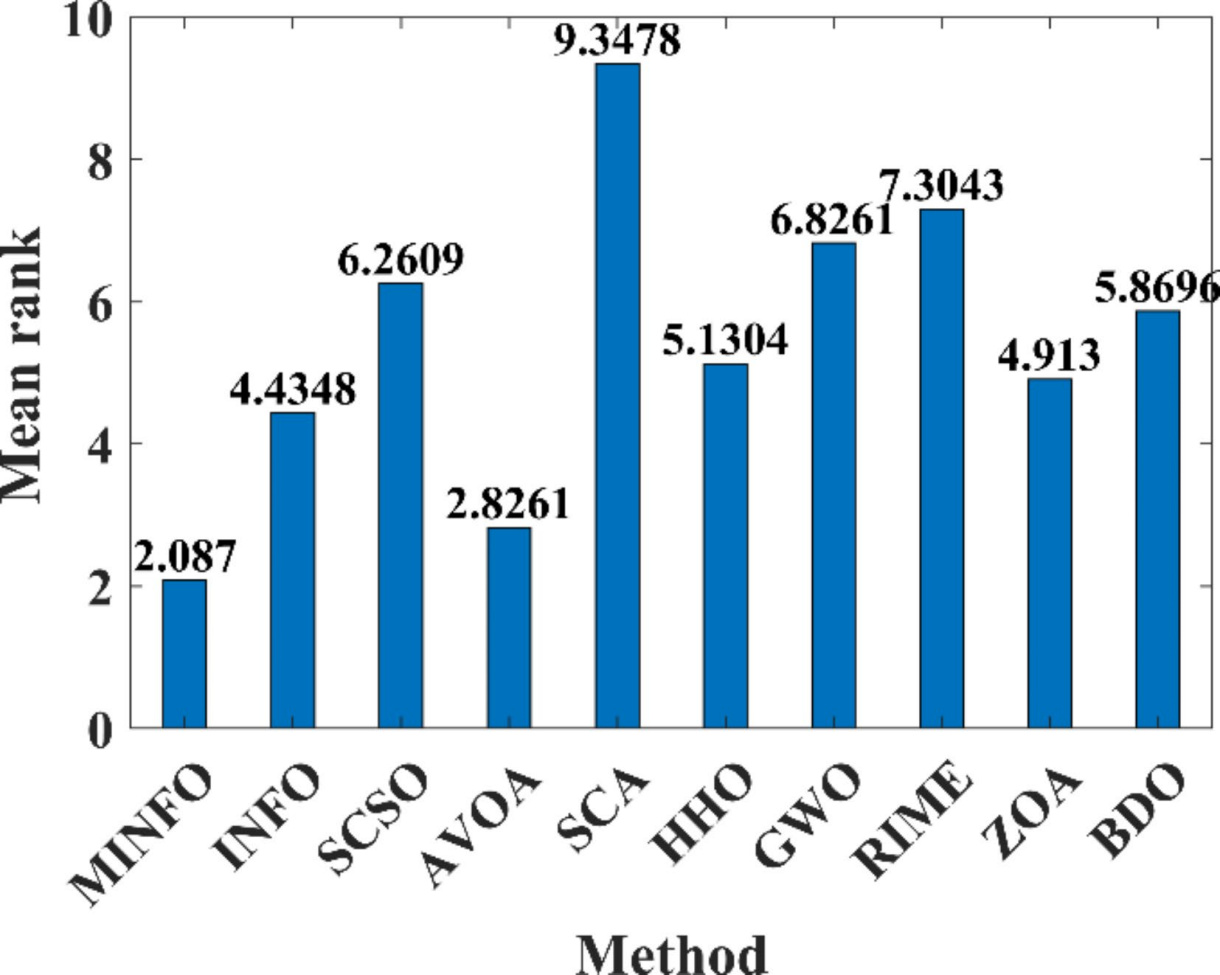
Friedman ranking for the traditional benchmark functions.

**Fig. 10:**
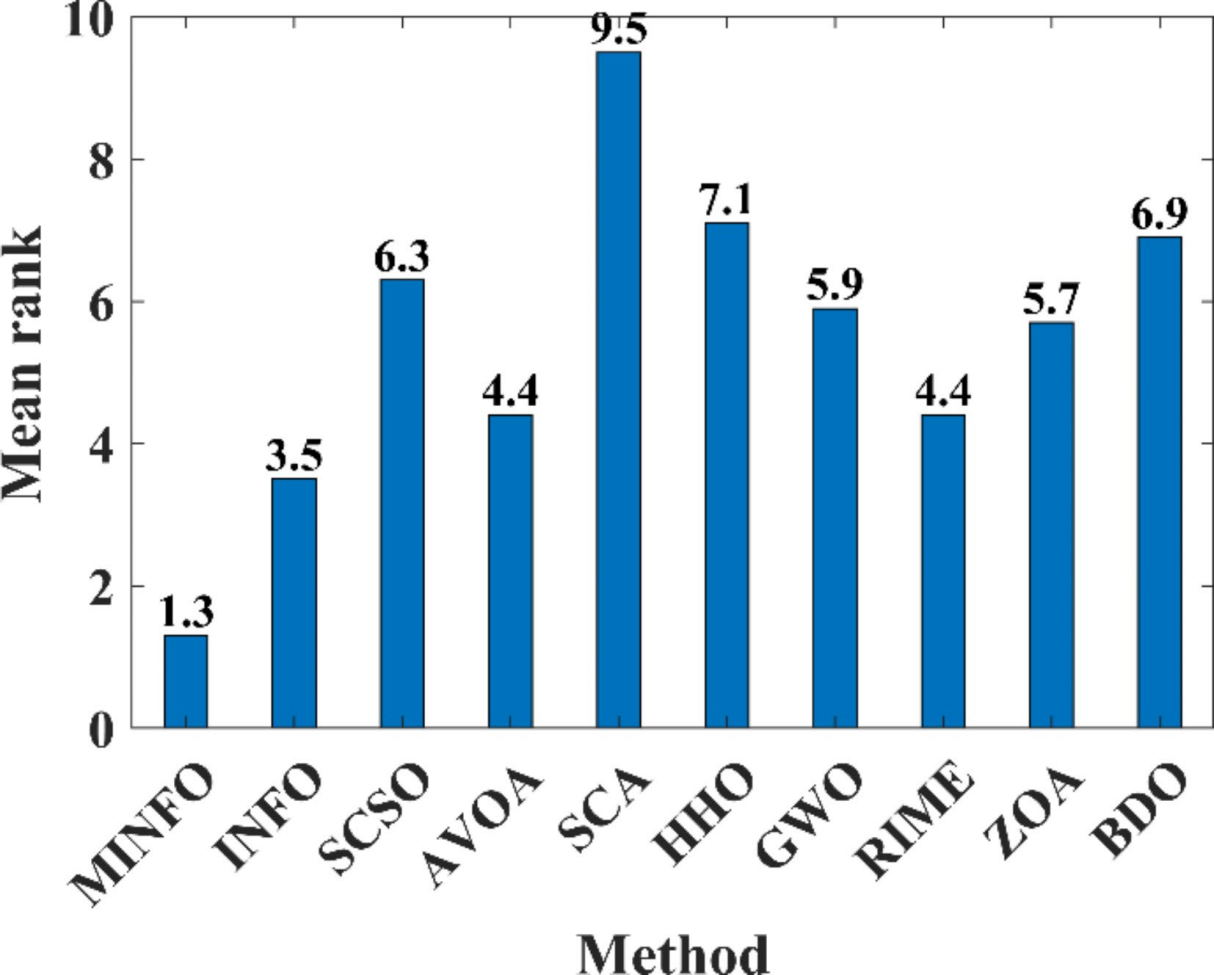
Friedman ranking for the CEC-2019 benchmark functions.

In this paper two statistical tests have been utilized including Wilcoxon rank sum and Friedman tests to assess the performance of the MINFO compared to the other reported optimization methods. The Wilcoxon rank sum test can be employed to compare the yielded results between two optimizers and verify if there is a significant difference between these optimizers which can be assigned using the *p*-value^46^. In the case of the *p*-value < 0.5, it verifies the significant difference between the optimizers’ outcomes is notable while in the case of *p*-value > 0.5, it verifies that there isn’t significant difference. Tables 4 and 5 tabulate the *p*-values between the suggested MINFO and the INFO, SCSO, AVOA, GWO, HHO, ZOA, WOA, RIME and BDO for the standard functions. As per the tabulated results in Tables 4 and 5, it is clear that the *p*-values < 0.5, i.e., there are significant differences between the MINFO and the other methods for most of the objective functions. However, for some cases, there are no significant differences between the MINFO and the other optimizers which depicted as bolded value where the *p*-values > 0.5. Likewise, Tables 6 and 7 tabulate the *p*-values between the suggested MINFO and the other techniques for the CEC-2019 test suite. Also, the *p*-values are less than 0.5 which means that there are notable differences between the yielded results from the MINFO and the other techniques for most cases. Except, the bolded values refer to that the *p*-values are more than 0.5 which refers to that there are no significant differences. Additionally, the NA (not available value) of *p*-value refers to the fact that the results can’t be compared because the results of the two optimizers are identical. Also, the Friedman’s test^47^ is employed to assess the performance of the MINFO against the other reported algorithms based on ranking the optimizers based on mean values of the obtained results. Figures 9 and 10 display the mean ranks of the comparative optimization methods for the basic and the CEC-2019 functions, respectively. Based on the performance of Friedman’s test, it is evident clear that the proposed MINFO is the best and it has minimum mean ranking compared to other methods.

**Table 4:** The *p*-values of MINFO vs. INFO, SCSO, AVOA, SCA and HHO for the standard benchmark functions.

MEEFO vs.	INFO	SCSO	AVOA	SCA	HHO
F1	8.0065E-09	8.0065E-09	8.0065E-09	8.0065E-09	8.0065E-09
F2	6.7956E-08	6.7956E-08	6.7956E-08	6.7956E-08	6.7956E-08
F3	8.0065E-09	8.0065E-09	8.0065E-09	8.0065E-09	8.0065E-09
F4	6.7956E-08	6.7956E-08	6.7956E-08	6.7956E-08	6.7956E-08
F5	1.1045E-05	6.7956E-08	6.7956E-08	6.7956E-08	6.7956E-08
F6	2.0616E-06	6.7956E-08	6.7956E-08	6.7956E-08	6.7956E-08
F7	6.7956E-08	4.6792E-02	3.0480E-04	6.7956E-08	5.6517E-02
F8	6.7956E-08	6.7956E-08	7.9479E-07	6.7956E-08	7.8980E-08
F9	NaN	NaN	NaN	8.01E-09	NaN
F10	NaN	NaN	NaN	8.01E-09	NaN
F11	NaN	NaN	NaN	8.01E-09	NaN
F12	0.0001444	6.7956E-08	1.2009E-06	6.7956E-08	6.0148E-07
F13	0.5427717	6.7956E-08	1.5997E-05	6.7956E-08	1.5997E-05
F14	0.1625869	8.0065E-09	3.2930E-07	8.0065E-09	8.0065E-09
F15	9.57E-06	8.9882E-07	1.1831E-06	5.1442E-07	1.1831E-06
F16	0.1553864	1.5149E-08	5.0032E-06	1.5149E-08	3.4286E-08
F17	NaN	8.0065E-09	3.4211E-01	8.0065E-09	8.0065E-09
F18	0.282781	1.5149E-08	1.5149E-08	1.5149E-08	1.5149E-08
F19	0.3421123	8.0065E-09	7.9919E-09	8.0065E-09	8.0065E-09
F20	0.1217252	0.0027488	0.5715402	2.64E-07	2.64E-07
F21	0.018418	3.9593E-08	3.9593E-08	3.9593E-08	3.9593E-08
F22	0.0286524	1.1267E-08	1.1267E-08	1.1267E-08	1.1267E-08
F23	0.0027538	2.4037E-08	2.4037E-08	2.4037E-08	2.4037E-08

**Table 5:** The *p*-values of MINFO vs. GWO, RIME, ZOA and BDO for the standard benchmark functions.

MEEFO vs.	GWO	RIME	ZOA	BDO
F1	8.0065E-09	8.0065E-09	8.0065E-09	8.0065E-09
F2	6.7956E-08	6.7956E-08	6.7956E-08	6.7956E-08
F3	8.0065E-09	8.0065E-09	8.0065E-09	8.0065E-09
F4	6.7956E-08	6.7956E-08	6.7956E-08	6.7956E-08
F5	6.7956E-08	6.7956E-08	6.7956E-08	6.7956E-08
F6	6.7956E-08	6.7956E-08	6.7956E-08	6.7956E-08
F7	6.7956E-08	6.7956E-08	4.9864E-02	1.3761E-06
F8	6.7956E-08	6.7956E-08	6.7956E-08	6.7956E-08
F9	7.95E-09	8.01E-09	NaN	NaN
F10	8.01E-09	8.01E-09	NaN	NaN
F11	0.019799	8.01E-09	NaN	NaN
F12	6.7956E-08	6.7956E-08	6.7956E-08	6.7956E-08
F13	1.0646E-07	1.8030E-06	6.7956E-08	6.7956E-08
F14	8.0065E-09	8.0065E-09	8.0065E-09	3.2846E-06
F15	6.8095E-07	4.4664E-07	1.1831E-06	9.6186E-07
F16	1.5149E-08	1.5149E-08	1.0675E-07	0.0003743
F17	8.0065E-09	8.0065E-09	9.4288E-06	NaN
F18	1.5149E-08	1.5149E-08	1.6425E-08	8.4971E-06
F19	8.0065E-09	8.0065E-09	8.0065E-09	2.9711E-07
F20	0.1189117	0.0227899	0.355187	0.0001001
F21	3.9593E-08	3.9593E-08	3.9593E-08	8.1771E-06
F22	1.1267E-08	1.1267E-08	1.1267E-08	2.3970E-05
F23	2.4037E-08	2.4037E-08	2.4037E-08	1.9262E-05

**Table 6:** The *p*-values of MINFO vs. INFO, SCSO, AVOA, SCA and HHO the CEC-2019 test suite.

MEEFO vs.	INFO	SCSO	AVOA	SCA	HHO
CEC01	1.99707E-04	6.79562E-08	6.79562E-08	6.79562E-08	6.79562E-08
CEC02	1.62449E-01	8.00655E-09	8.00655E-09	8.00655E-09	8.00655E-09
CEC03	1.62449E-01	8.00655E-09	2.98679E-08	8.00655E-09	8.00655E-09
CEC04	2.94409E-02	2.04071E-05	7.57738E-06	6.79562E-08	2.56295E-07
CEC05	8.18149E-01	2.95975E-07	1.03734E-04	6.79562E-08	6.79562E-08
CEC06	2.56393E-02	1.78238E-03	5.79218E-01	7.89803E-08	4.54008E-06
CEC07	1.01410E-03	7.94795E-07	9.20913E-04	6.79562E-08	5.22689E-07
CEC08	2.13926E-03	5.16578E-06	1.57567E-06	9.17277E-08	2.95975E-07
CEC09	2.47061E-04	6.79562E-08	1.91771E-07	6.79562E-08	1.23464E-07
CEC10	1.22718E-03	2.68977E-06	4.24883E-01	6.79562E-08	2.21776E-07

**Table 7:** The *p*-values of MINFO against GWO, RIME, ZOA and BDO for the CEC-2019 test suite.

MEEFO vs.	GWO	RIME	ZOA	BDO
CEC01	6.79562E-08	6.79562E-08	6.79562E-08	1.91771E-07
CEC02	8.00655E-09	8.00655E-09	8.00655E-09	2.35922E-05
CEC3	8.00655E-09	8.00655E-09	8.00655E-09	6.51599E-05
CEC4	3.50702E-01	6.01106E-02	1.43085E-07	2.28694E-01
CEC5	3.49946E-06	2.22699E-02	7.89803E-08	8.29242E-05
CEC6	6.79562E-08	4.38804E-02	1.44383E-04	1.43085E-07
CEC7	1.10447E-05	2.97677E-01	2.28694E-01	7.89803E-08
CEC8	3.38195E-04	4.15502E-04	3.74990E-04	7.89803E-08
CEC9	6.79562E-08	2.30247E-05	6.79562E-08	3.04799E-04
CEC10	1.04727E-06	1.99707E-04	1.29405E-04	2.56295E-07

As per the obtained results on the standard and CEC 2019 benchmark functions, the specific performance improvement is that the proposed MINFO has a robust exploitation performance according to the unimodal functions (F1-F7). In addition to that it has a robust exploration performance according to the multimodal functions (F8 - F16) and it can balance between the exploration and exploitation performance according to composition functions (F17 - F23). Furthermore, the suggested optimizer has fast convergence characteristics compared to other optimizers.

### Solving the smart home

**Fig. 11:**
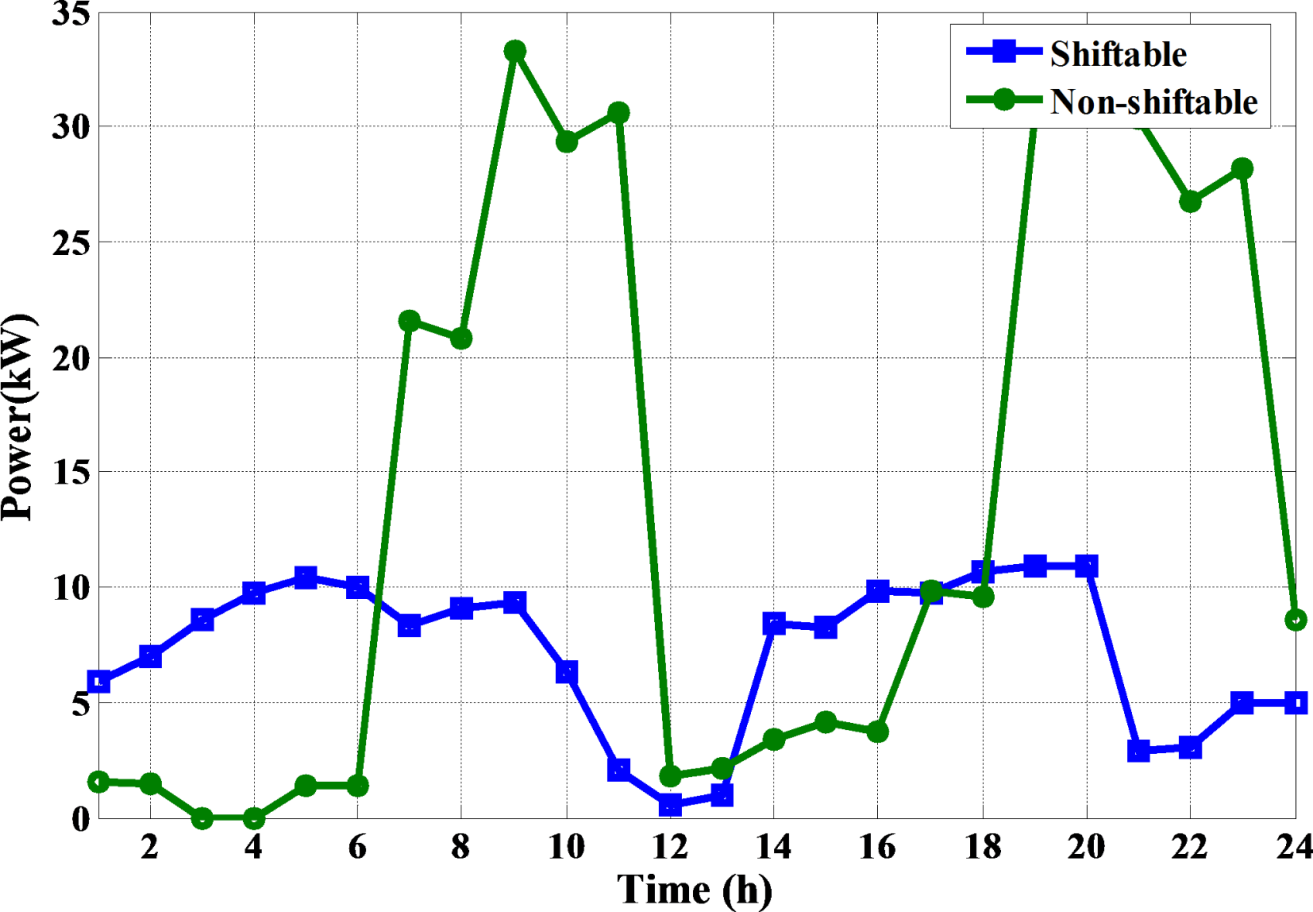
The load pattern during the day ahead.

**Fig. 12:**
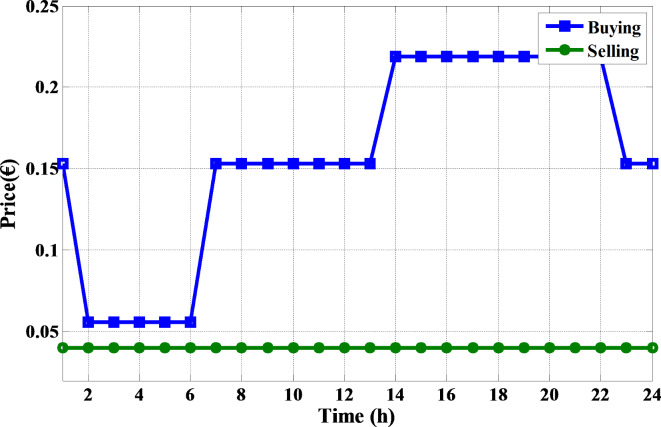
The buying and selling cost of energy.

The proposed MINFO is employed to solve EM of the smart building for reducing the bill cost, and the PAR separately and concurrently. Also, for fair comparison the obtained results were compared to INFO, SCSO, AVOA, GWO, HHO, ZOA, WOA, RIME and BDO. The parameters of all optimization methods are selected the same as Table 1 except the populations and iterations numbers are 25, and 200, respectively. The study building is a medium-sized hotel which is composed of shiftable and non-shiftable loads. The power supplied to the loads via the grid, PV panel, barriers unit and two diesel generators The rating of the PV unit, the first diesel, the second diesel, the capacity and maximum charging/discharging power of the batteries unit are 15 kW, 30 kW, 10 kW, 47.5 kWh and 7.6 kW, respectively^48^. The allowable injected or absorbed power from grid is in the range of [-30, 30] kW. Figure 11 displays the profile of the shiftable and non-shiftable loads. The buying and selling energy cost from or to grid are depicted in Fig. 12 While the cost coefficients including $\:{K}^{D}$, $\:{K}^{PV}$, and $\:{K}^{Battery}$ are selected to be 0.5, 0 and 0.05 €/kWh, respectively^48^. The purpose of application the DSR is to reduce the bill cost which can be accomplished by shifting the loads from the high price periods to low price periods. Consequently, the bill will be reduced considerably.

#### Case 1 solving the EM of the smart home for cost reduction

**Fig. 13:**
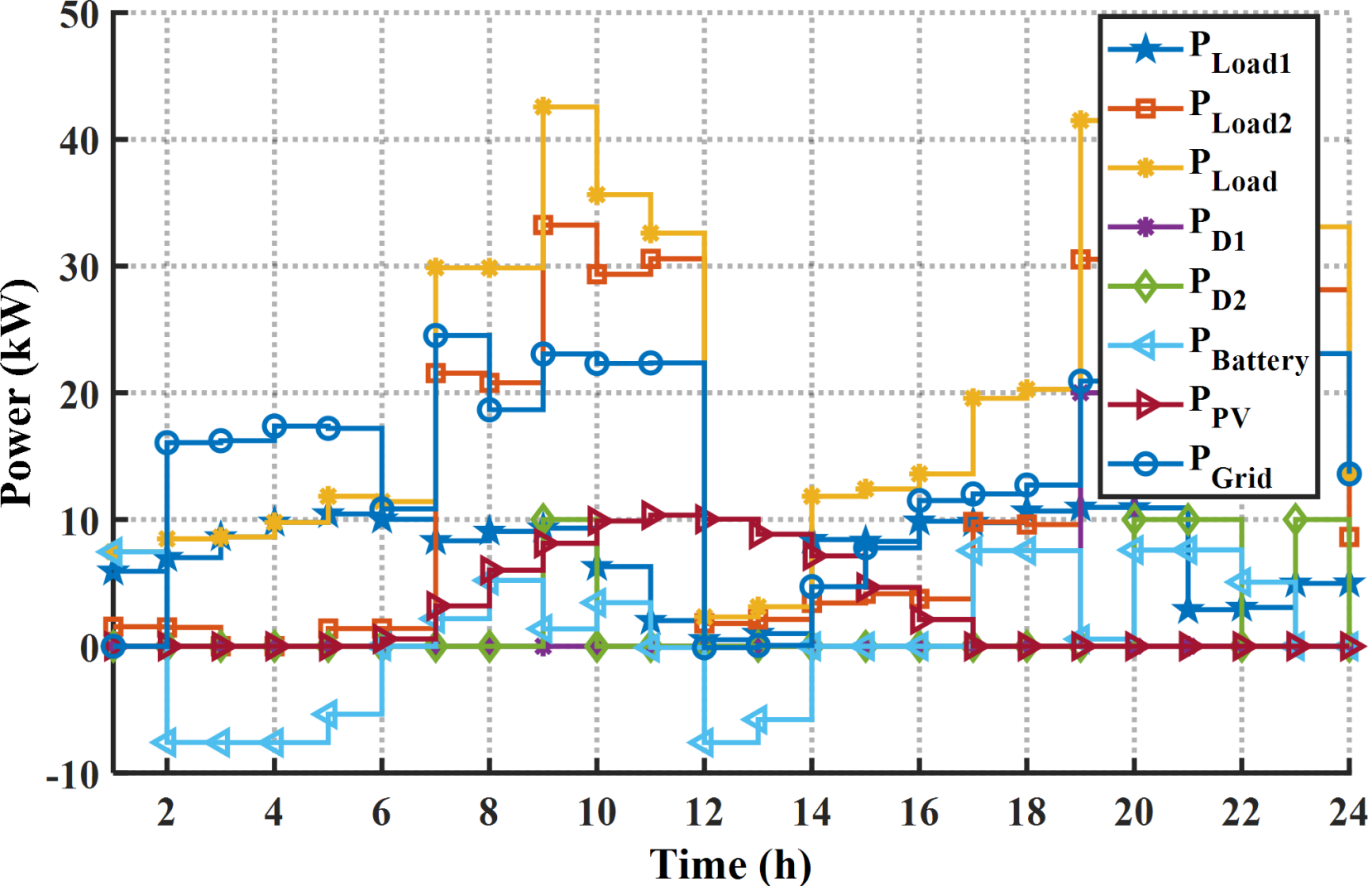
The optimal schedule of units for bill cost reduction without DSR.

In this case the EM of the smart building is solved using the suggested MINFO for reducing the bill cost of energy. Initially, without EM solution (base case) the bill cost for the whole day is 169.96 € while the PAR is 2.024 p.u. In the case of optimal scheduling the energy sources in this building without DSR the cost is reduced to 86.50 € or by 49.1%. Figure 13; Table 8 display the optimal scheduling of the generation units of the smart building for cost reduction without the DSR. According to Fig. 13; Table 8, the output power of the PV system follows variation of solar irradiance during the day ahead. The battery starts charging at the off-peak period in which the purchasing energy cost is low, and it discharges at the on-peak periods. According to the results in Fig. 13; Table 8, the diesel generators start work when the total power of the load demand is more than 30 kW. Table 9 tabulates the worst, the best and average values of the total costs that have been obtained by other techniques. From Table 9, the superior results have been obtained by MINFO application in which the best cost of MINFO is less than the INFO, SCSO, AVOA, GWO, HHO, ZOA, WOA, RIME and DBO by 7.3%, 3.21%, 3.44%, 9.96%, 23.94%, 1.86%, 3.17%, 20.28%, and 14.34%, respectively.

**Fig. 14:**
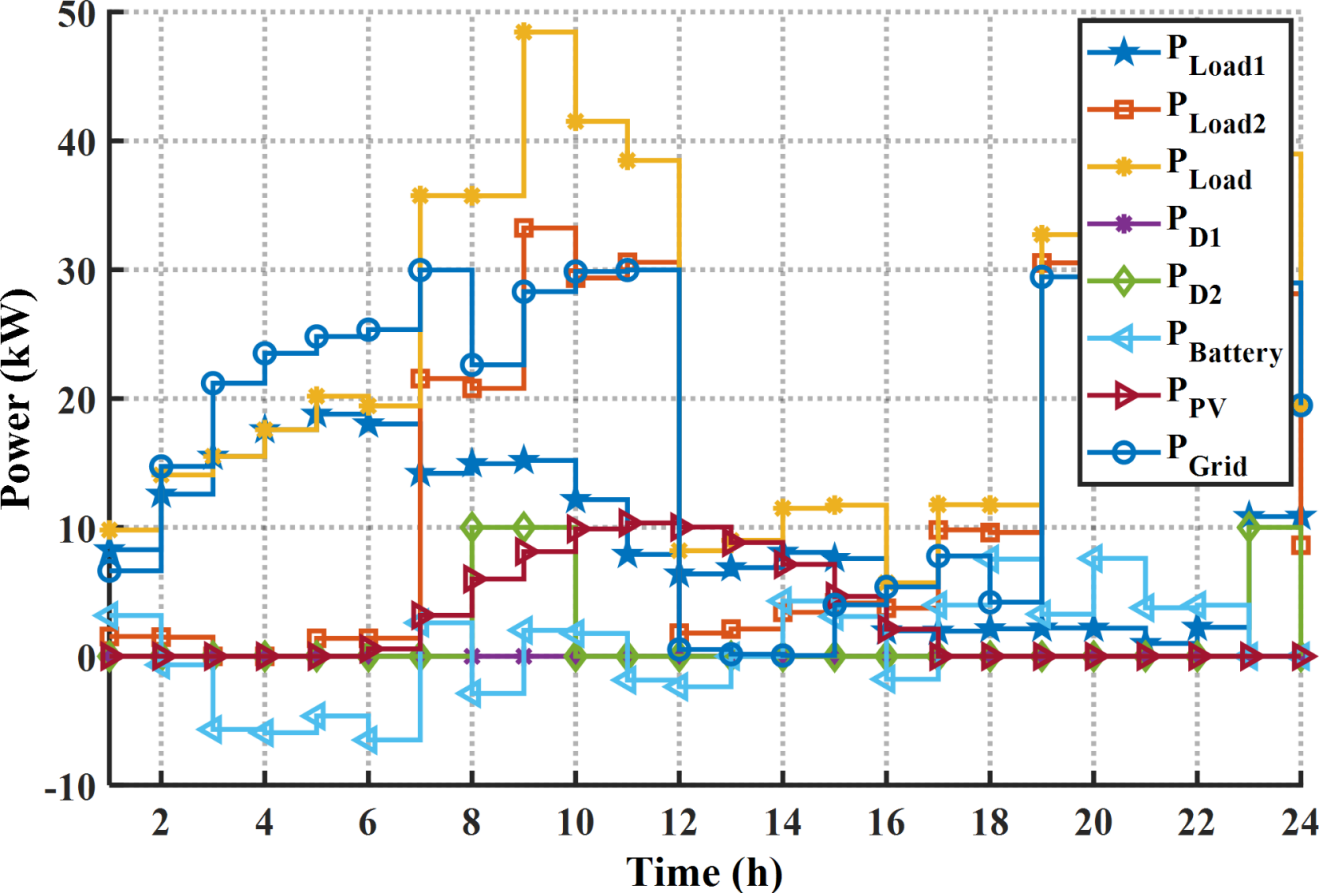
The optimal schedule of units for bill cost reduction with DSR.

**Fig. 15:**
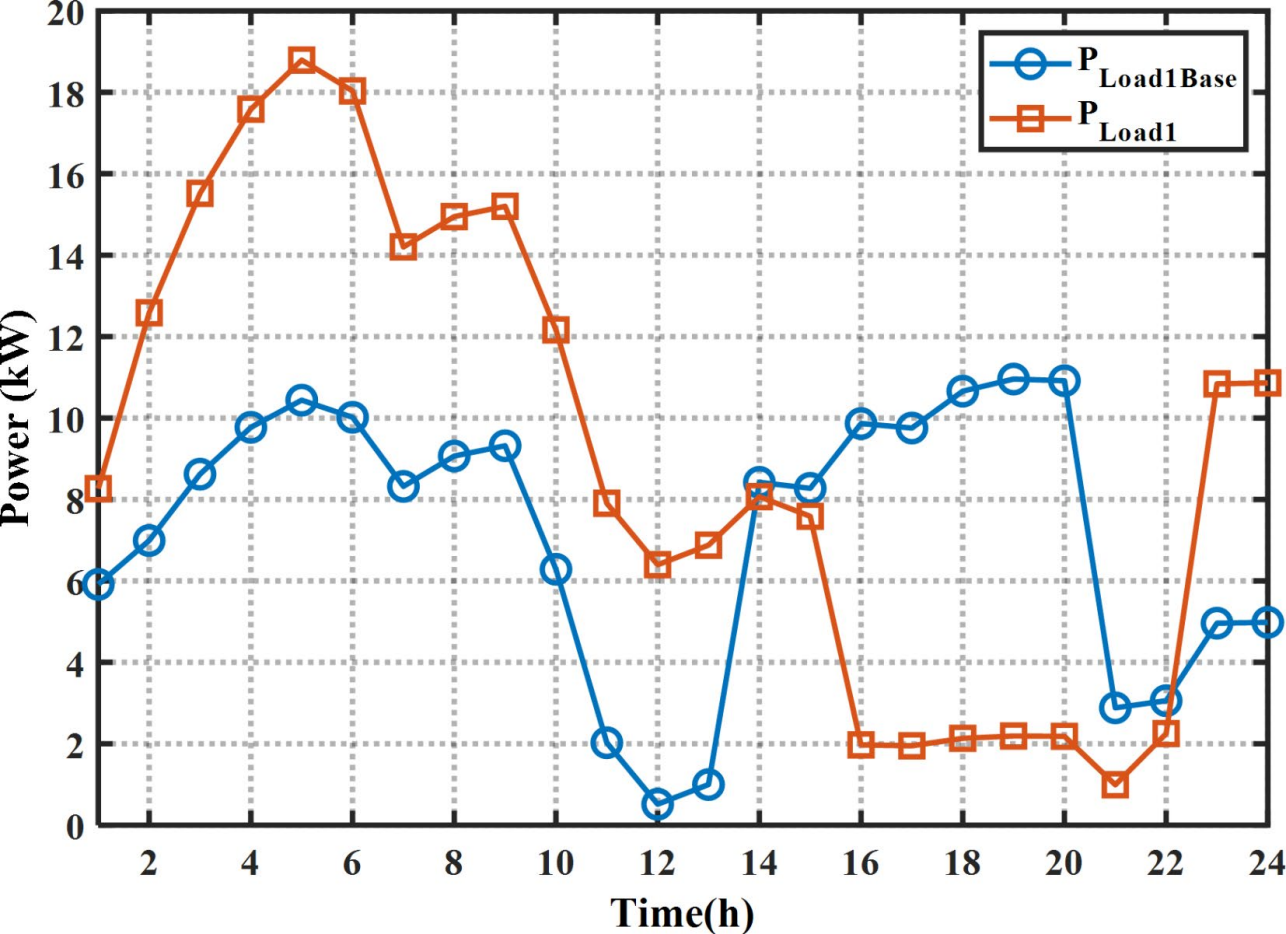
The Load demand of the shiftable loads after (base case) and before DSR for cost reduction.

In case of EM solution of the smart building for cost reduction with application the DSR, the cost is reduced to 79.55 $ or by 53.20% compared to the base case or by 8.4% compared to without application the DSR. The optimal schedule of the system components for this case is provided in Fig. 14; Table 10. As per the provided scheduling results, At the first 12 h, the smart home draws high power from the grid and the batteries system starts charging in which the price of the energy is low. Additionally, due to application of the DSR, only the second diesel generator is on while the other diesel generator is off during the day ahead. The statistical results of this case are provided in Table 11. It is evident clear that the minimum cost can be obtained by application of the MINFO in which the best cost of MINFO is less than the INFO, SCSO, AVOA, GWO, HHO, ZOA, WOA, RIME and BDO by 10.79%, 11.00%, 22.3%, 17.63%, 40.9%, 3.62%, 7.21%, 29.84%, and 13.22%, respectively. The load pattern of the shiftable loads after and before application the DSR program is shown in Fig. 15. It is obvious that the loads are shifted away from the on-peak periods to other peak periods.

**Table 8:** The optimal schedule of units for bill cost reduction without DSR.

Hour	$P_{Load1}$	$P_{Load2}$	$P_{Load}$	$P_{D1}$	$P_{D2}$	$P_{Battery}$	$P_{PV}$	$P_{Grid}$
1	5.91	1.54	7.45	0	0	7.45	0	0.00
2	6.99	1.49	8.48	0	0	-7.59	0	16.7
3	8.62	0	8.62	0	0	-7.60	0	16.22
4	9.77	0	9.77	0	0	-7.60	0	17.37
5	10.44	1.39	11.83	0	0	-5.36	0	17.20
6	10.02	1.40	11.42	0	0	0	0.58	10.84
7	8.32	21.55	29.87	0	0	2.17	3.18	24.52
8	9.7	20.79	29.86	0	0	5.19	6.00	18.67
9	9.32	33.24	42.56	0	10	1.37	8.12	23.7
10	6.29	29.36	35.64	0	0	3.44	9.88	22.32
11	2.3	30.58	32.60	0	0	-0.10	10.34	22.36
12	0.52	1.80	2.32	0	0	-7.60	10.3	(0.11)
13	1.00	2.12	3.11	0	0	-5.79	8.83	0.7
14	8.42	3.41	11.83	0	0	0	7.13	4.70
15	8.27	4.14	12.42	0	0	0	4.65	7.77
16	9.87	3.74	13.60	0	0	0	2.10	11.50
17	9.76	9.81	19.57	0	0	7.54	0.00	12.02
18	10.66	9.61	20.27	0	0	7.54	0	12.73
19	10.96	30.53	41.49	20	0	0.57	0	20.92
20	10.92	31.26	42.18	0	10	7.59	0	24.59
21	2.88	30.38	33.27	0	10	7.60	0	15.67
22	3.6	26.74	29.80	0	0	5.6	0	24.74
23	4.96	28.14	33.10	0	10	0	0	23.10
24	4.98	8.62	13.61	0	0	0	0	13.61

**Table 9:** The statistical outcome for cost reduction without DSR.

Algorithm	Average	Best	Worst	SD
MINFO	96.80	86.50	106.79	4.78
INFO	103.42	93.4	113.26	5.22
SCSO	98.83	89.37	111.42	5.42
AVOA	104.31	89.58	119.56	6.56
SCA	38,424	96.7	448,002	123,537
HHO	132.66	113.72	155.18	10.42
GWO	97.75	88.14	14.93	4.62
RIME	98.11	89.33	111.62	5.93
ZOA	143.02	18.50	172.12	18.42
DBO	24,356.91	100.98	517,567.48	14,258.65

**Table 10:** The optimal schedule of units for bill cost reduction with DSR.

Hour	$P{Load1}$	$P{Load2}$	$P_{Load}$	$P_{D1}$	$P_{D2}$	$P_{Battery}$	$P_{PV}$	$P_{Grid}$
1	8.27	1.54	9.80	0	0	3.16	0.00	6.64
2	12.58	1.49	14.7	0	0	-0.66	0.00	14.73
3	15.52	0.00	15.52	0	0	-5.68	0.00	21.20
4	17.58	0.00	17.58	0	0	-5.93	0.00	23.52
5	18.79	1.39	20.19	0	0	-4.63	0.00	24.82
6	18.3	1.40	19.44	0	0	-6.50	0.58	25.35
7	14.20	21.55	35.75	0	0	2.60	3.18	29.98
8	14.95	20.79	35.74	0	10	-2.88	6.00	22.62
9	15.20	33.24	48.44	0	10	2.02	8.12	28.30
10	12.17	29.36	41.52	0	0	1.78	9.88	29.86
11	7.91	30.58	38.48	0	0	-1.84	10.34	29.98
12	6.40	1.80	8.20	0	0	-2.36	10.3	0.53
13	6.88	2.12	8.99	0	0	0.00	8.83	0.16
14	8.7	3.41	11.48	0	0	4.29	7.13	0.6
15	7.59	4.14	11.73	0	0	3.9	4.65	3.99
16	1.97	3.74	5.71	0	0	-1.78	2.10	5.38
17	1.95	9.81	11.76	0	0	3.98	0.00	7.78
18	2.13	9.61	11.74	0	0	7.54	0.00	4.20
19	2.19	30.53	32.72	0	0	3.27	0.00	29.45
20	2.18	31.26	33.45	0	0	7.60	0.00	25.85
21	0.99	30.38	31.38	0	0	3.77	0.00	27.61
22	2.25	26.74	28.99	0	0	3.97	0.00	25.02
23	10.84	28.14	38.98	0	10	0.00	0.00	28.98
24	10.86	8.62	19.49	0	0	0.00	0.00	19.49

**Table 11:** The statistical outcomes for cost reduction with DSR.

Algorithm	Mean	Best	Worst	SD
MINFO	94.72	79.55	15.9	6.76
INFO	102.16	89.17	133.19	11.5
SCSO	13.69	89.38	119.42	9.51
AVOA	130.88	102.3	192.68	20.74
SCA	110.70	96.58	124.87	7.68
HHO	175.59	132.79	218.72	22.18
GWO	97.73	82.54	116.14	10.16
RIME	97.89	85.73	19.62	7.8
ZOA	143.38	113.39	177.53	19.54
DBO	117.19	91.67	194.90	22.71

#### Case 2 solving the EM of the smart home for PAR

**Fig. 16:**
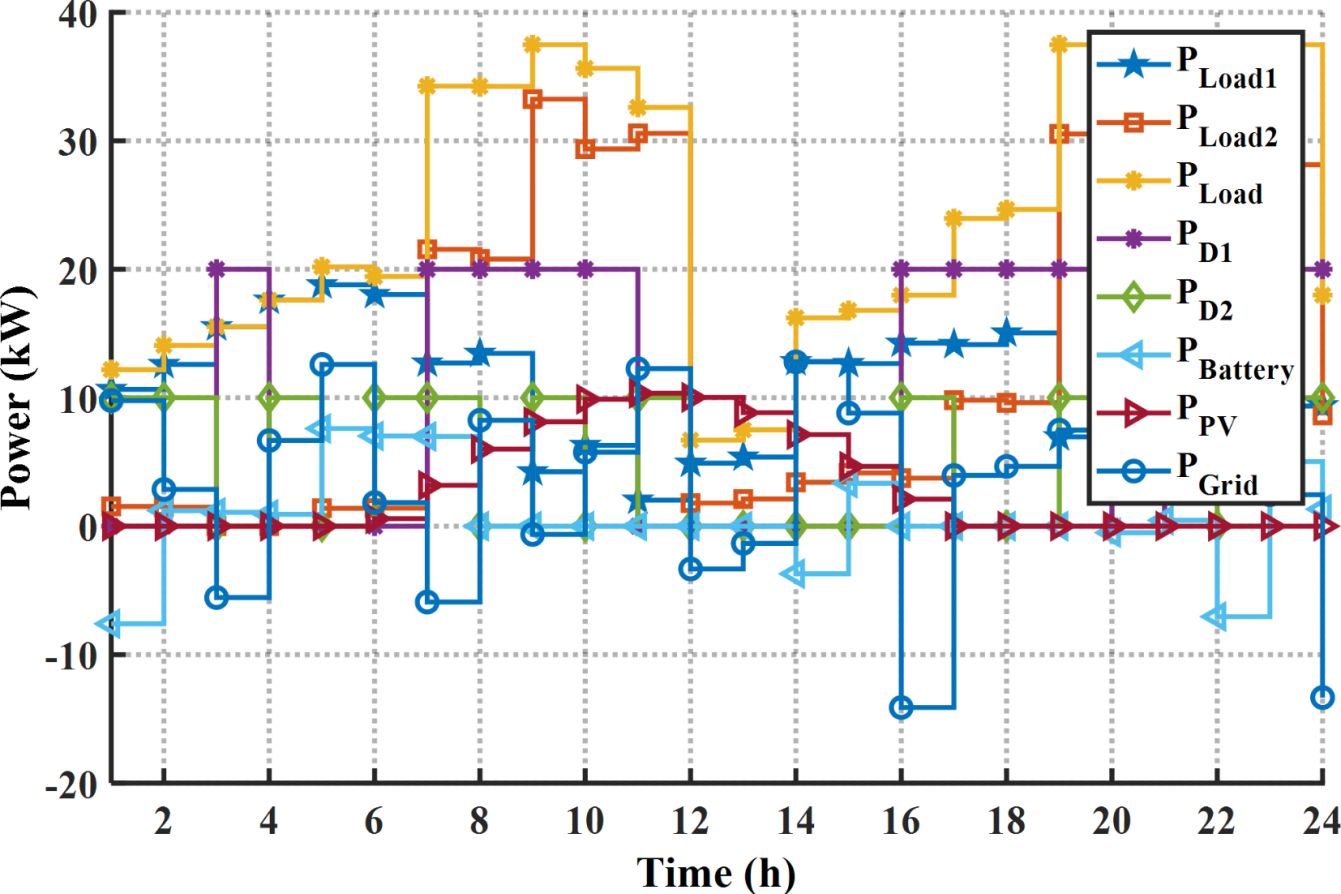
The optimal schedule for PAR reduction.

**Fig. 17:**
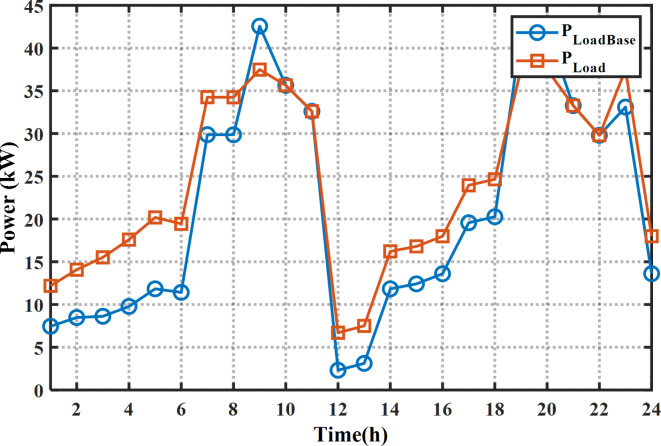
The Load demand after and before DSR for PAR reduction.

In this case, the EM of the smart building has been solved for reducing the PAR using the proposed MINFO. In the base case the PAR value is the PAR is 2.024 p.u. while in the case of solving the EM by the MINFO the minimum obtained PAR is 1.5496 p.u. In other words, the PAR is reduced by 53.19% compared to base case. As per the scheduling results in Fig. 16; Table 12, the two diesel generators work for many hours to provide the power to the shifted load as well as the battery. The load of the building after and before the DSR is displayed in Fig. 17. As per the displayed load pattern, the maximum load at base case was 42.56 kW and it has been decreased to 37.48 kW. Likewise, the load demands at time 19:00 and 20:00 p.m. have decreased from 41.49 kW, and 42.18 kW to 37.48 kW and 37.48 kW, respectively. Table 13 lists the statistical outcome for PAR reduction. According to Table 13, the minimum PAR can be obtained by application the MINFO and INFO.

**Table 12:** The optimal schedule of EM solution for the PAR reduction.

Hour	$P_{Load1}$	$P_{Load2}$	$P_{Load}$	$P_{D1}$	$P_{D2}$	$P_{Battery}$	$P_{PV}$	$P_{Grid}$
1	10.65	1.54	12.19	0	10	-7.60	0.00	9.78
2	12.58	1.49	14.7	0	10	1.21	0.00	2.86
3	15.52	0.00	15.52	20	0	1.7	0.00	-5.55
4	17.59	0.00	17.59	0	10	0.92	0.00	6.67
5	18.79	1.39	20.19	0	0	7.60	0.00	12.59
6	18.3	1.40	19.44	0	10	7.02	0.58	1.83
7	12.70	21.55	34.26	20	10	6.98	3.18	-5.90
8	13.45	20.79	34.24	20	0	0.00	6.00	8.24
9	4.24	33.24	37.48	20	10	0.00	8.12	-0.64
10	6.29	29.36	35.64	20	0	0.00	9.88	5.76
11	2.3	30.58	32.60	0	10	0.00	10.34	12.26
12	4.90	1.80	6.70	0	0	0.00	10.3	-3.33
13	5.38	2.12	7.50	0	0	0.00	8.83	-1.34
14	12.80	3.41	16.22	0	0	-3.70	7.13	12.79
15	12.66	4.14	16.80	0	0	3.34	4.65	8.81
16	14.25	3.74	17.98	20	10	0.00	2.10	-14.12
17	14.14	9.81	23.95	20	0	0.00	0.00	3.95
18	15.4	9.61	24.65	20	0	0.00	0.00	4.65
19	6.95	30.53	37.48	20	10	0.00	0.00	7.48
20	6.22	31.26	37.48	0	10	-0.50	0.00	27.99
21	2.88	30.38	33.27	20	10	0.46	0.00	2.81
22	3.6	26.74	29.80	20	0	-7.4	0.00	16.84
23	9.34	28.14	37.48	20	10	5.3	0.00	2.45
24	9.37	8.62	17.99	20	10	1.33	0.00	-13.34

**Table 13:** The statistical outcomes for PAR reduction.

Algorithm	Mean	Best	Worst	SD
MINFO	1.5496	1.5496	1.5496	0.0000
INFO	1.5496	1.5496	1.5496	0.0000
SCSO	1.576	1.558	1.657	0.0143
AVOA	1.5666	1.5525	1.5963	0.0120
SCA	1.6163	1.5996	1.6443	0.0123
HHO	1.5793	1.5622	1.605	0.018
GWO	1.5593	1.559	1.5848	0.0100
RIME	1.5517	1.5497	1.5551	0.0015
ZOA	1.5736	1.563	1.5993	0.086
DBO	1.588	1.5616	1.6028	0.0112

#### Case 3. Solving the EM of the smart building for cost and PAR

**Fig. 18:**
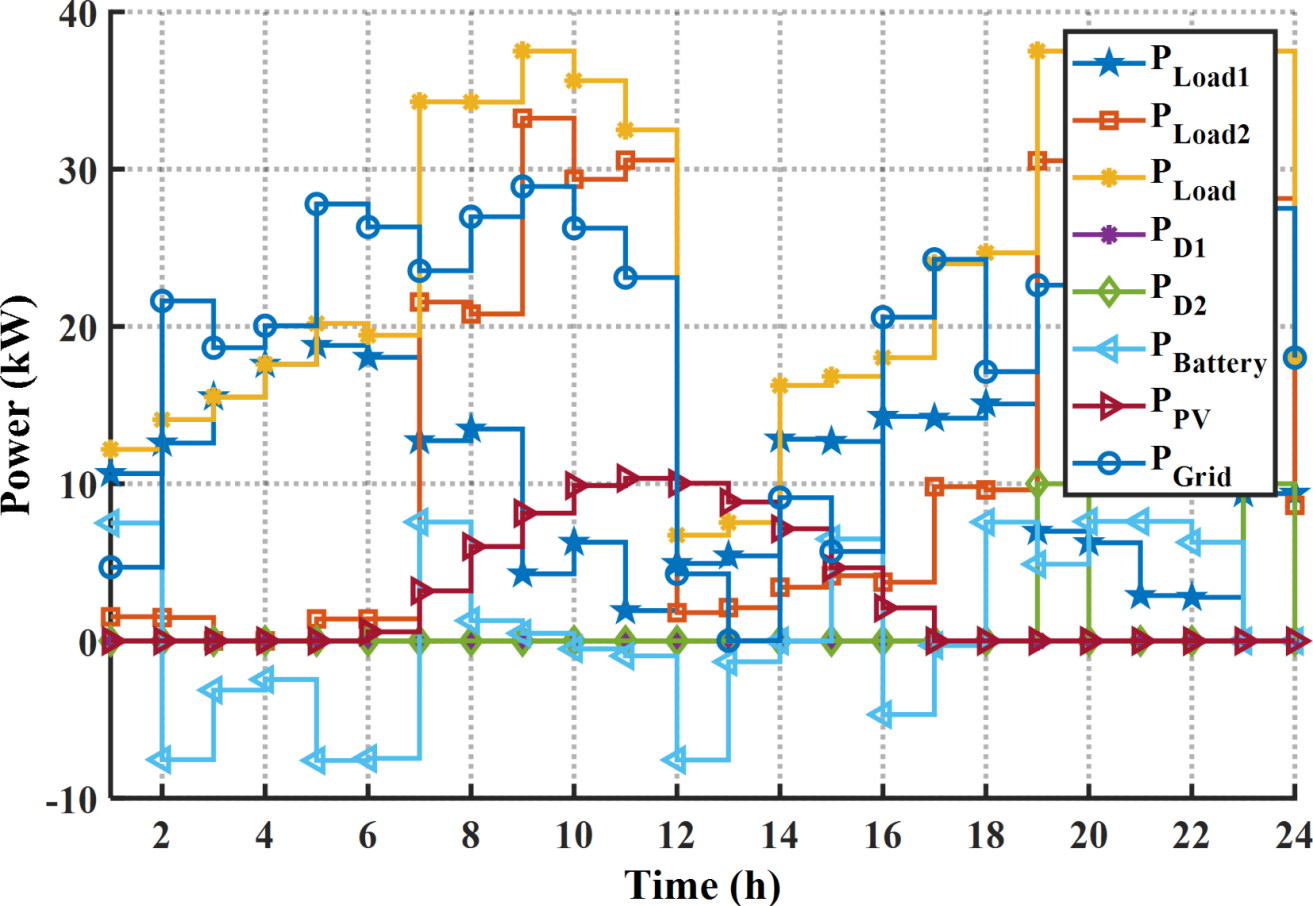
The optimal schedule for cost and PAR reduction.

**Fig. 19:**
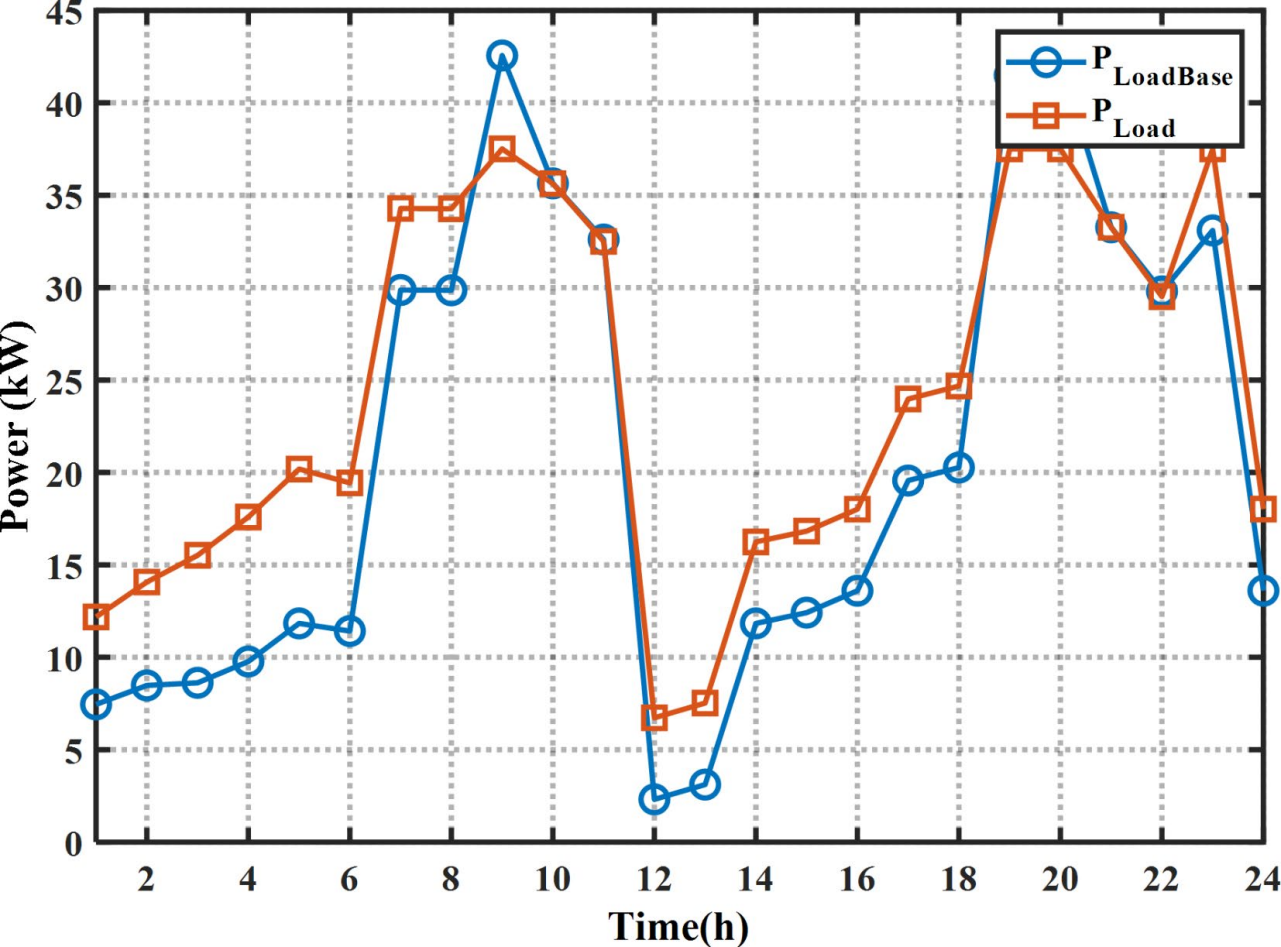
The Load demand after and before application the DSR for cost and PAR reduction.

**Fig. 20:**
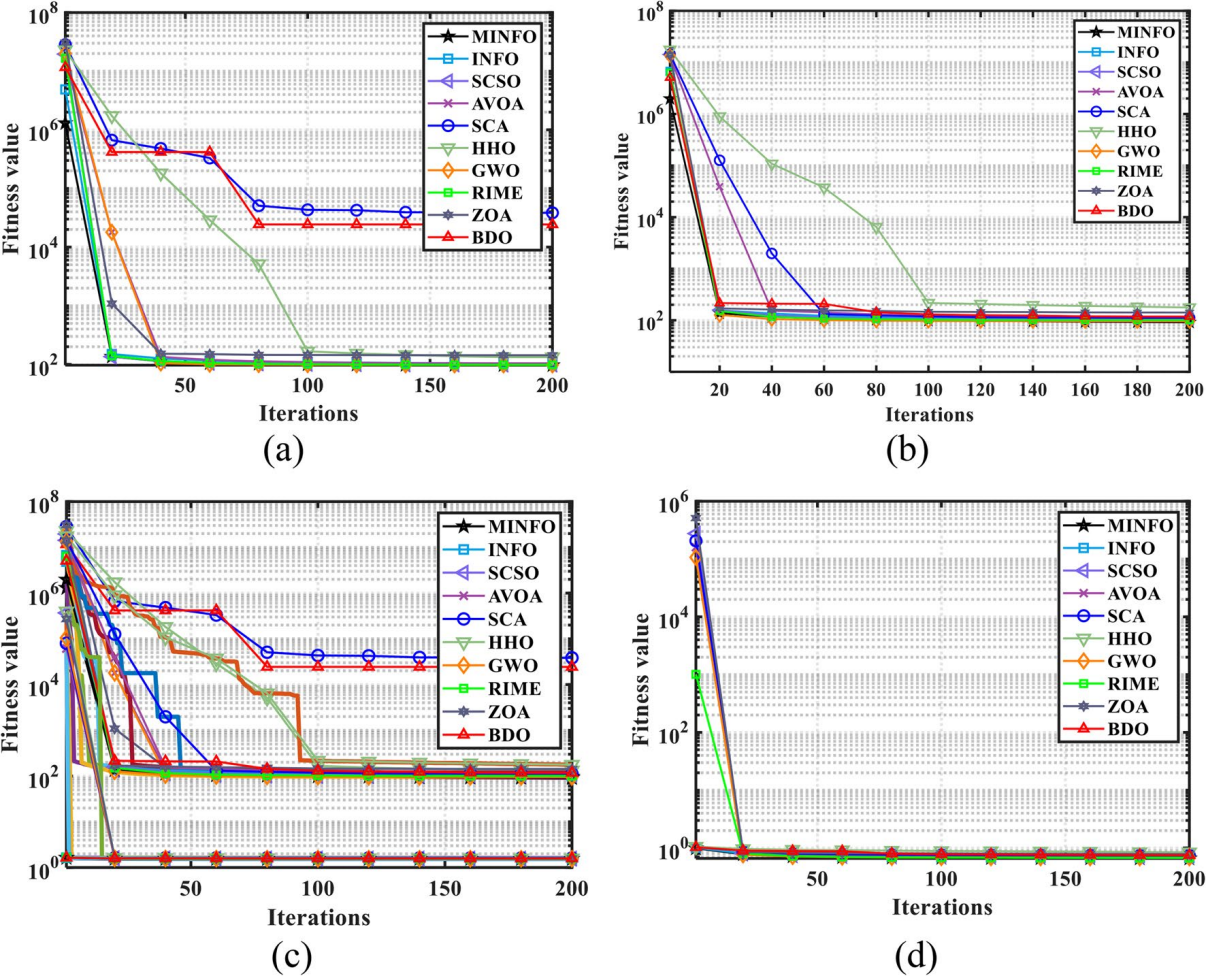
The convergence carves of the MINFO and the other optimizers for **a** Case 1 without DSR, **b** Case 1 with DSR, **c** Case 2, **d** Case 3.

Here, the cost and PAR are optimized concurrently by application of the suggested MINFO. The optimal cost and PAR obtained by EM solution are 83.55 $, and 1.558 p.u., respectively. In other words, the cost and PAR were reduced by 50.84% and 23.38%, respectively. The optimal scheduling for this case is depicted in Fig. 18; Table 14. It is clear that first diesel is not working while the second diesel generator worked for two hours only, this is due to the generation cost from the diesel generators is high. Additionally, the battery is charged at the off-peak and mid-peak periods while it is discharged at the on-peak period. Figure 19 displays the pattern of the load demand after and before DSR for cost and PAR minimization. The maximum load at base case was 42.56 kW and it has been decreased to 37.51 kW. Likewise, the load demands at time 19:00 and 20:00 p.m. have decreased from 41.49 kW, and 42.18 kW to 37.51 kW and 37.51 kW, respectively. Table 15 lists the statistical outcome for this case. It is clear that the MINFO is superior compared to other techniques. Finally, the trend of the convergences for all cases is shown in Fig. 20. It is clear that the proposed MINFO converged rapidly to the best solution for all studied cases. Finally, as per the comprehensive survey, the contribution of the paper to the field of smart building energy management in comparison to other existing optimization methods is that the energy management is solved not only with application the DSR for the appliance but also it presented an optimal scheduling of the energy sources to reduce the cost considerably.

**Table 14:** The optimal schedule of EM solution for cost and PAR reduction.

Hour	$P_{Load1}$	$P_{Load2}$	$P_{Load}$	$P_{D1}$	$P_{D2}$	$P_{Battery}$	$P_{PV}$	$P_{Grid}$
1	10.65	1.54	12.19	0	0	7.50	0	4.69
2	12.59	1.49	14.7	0	0	− 7.55	0	21.62
3	15.52	0	15.52	0	0	− 3.13	0	18.65
4	17.59	0	17.59	0	0	− 2.45	0	20.4
5	18.79	1.40	20.19	0	0	-7.60	0	27.79
6	18.3	1.40	19.44	0	0	− 7.47	0.58	26.33
7	12.73	21.55	34.29	0	0	7.56	3.18	23.54
8	13.48	20.79	34.27	0	0	1.29	6.00	26.98
9	4.27	33.24	37.51	0	0	0.49	8.12	28.90
10	6.27	29.36	35.63	0	0	-0.51	9.88	26.25
11	1.92	30.58	32.50	0	0	-0.95	10.35	23.11
12	4.93	1.80	6.73	0	0	-7.57	10.3	4.27
13	5.41	2.12	7.53	0	0	- 1.33	8.83	0.02
14	12.83	3.41	16.25	0	0	0	7.13	9.11
15	12.69	4.14	16.83	0	0	6.49	4.65	5.69
16	14.28	3.74	18.01	0	0	− 4.68	2.10	20.59
17	14.17	9.81	23.98	0	0	-0.29	0.00	24.27
18	15.7	9.61	24.68	0	0	7.56	0	17.12
19	6.97	30.53	37.51	0	10.00	4.88	0	22.63
20	6.25	31.26	37.51	0	0	7.60	0	29.91
21	2.88	30.38	33.27	0	0	7.60	0	25.67
22	2.77	26.74	29.51	0	0	6.27	0	23.24
23	9.37	28.14	37.51	0	10.00	0	0	27.51
24	9.39	8.62	18.02	0	0	0	0	18.02

**Table 15:** The statistical outcomes for cost and PAR reduction.

Algorithm	Mean	Best	Worst	SD
MINFO	0.665	0.6289	0.6754	0.0116
INFO	0.6740	0.6388	0.7219	0.0227
SCSO	0.7138	0.6627	0.7595	0.0255
AVOA	0.7144	0.6656	0.8255	0.354
SCA	0.7394	0.713	0.7736	0.0127
HHO	0.8435	0.7822	0.9263	0.48
GWO	0.6780	0.6528	0.7160	0.0193
RIME	0.6765	0.6467	0.768	0.0162
ZOA	0.7831	0.6974	0.8602	0.427
DBO	0.7494	0.6910	0.8280	0.344

## Conclusion

In this paper a modified Weighted mean of vectors (MINFO) algorithm was developed for solving the energy management of a smart building. Two modifications were applied to the suggested optimizer including the ECQOBL and the ALFM to enhance its global searching accuracy. The total cost and PAR have been optimized in the proposed EM solution with TOU based DSR and optimal scheduling of the energy resources. The salient findings of this research can be concluded as follows:

- The proposed MINFO is an efficient optimization method for solving the energy management solution.
- The MINFO has a stable performance and the best global searching accuracy which have been demonstrated via convergence trends, statistical comparisons, boxplots, Wilcoxon rank sum and Friedman tests on 33 benchmark functions.
- The minimum cost and PAR can be achieved by the proposed MINFO compared to INFO, SCSO, AVOA, GWO, HHO, ZOA, WOA, RIME and DBO.
- In the case of solving the EM for the cost only without application DSR, the cost was reduced from 169.96 € to 86.50 € or by 49.1% compared to the base case.
- In the case of solving the EM for the cost only, the cost was reduced from 169.96 € to 79.55 € or by 53.20% compared to the base case.
- In the case of solving the EM for the cost and PAR simultaneously, the cost was reduced to 83.55 $ (50.84%) and PAR was reduced to 1.558 p.u (23.38%) compared to the base case.

The limitations of the proposed algorithm for energy management in smart buildings are that uncertainties of the electric system weren’t considered in energy management solutions of the smart homes. Thus, future work will focus on solving energy management in smart buildings with considering the uncertainties of load demand and the renewable energy sources.

## Data Availability

The datasets generated during and/or analyzed during the current study are available from the corresponding author on reasonable request.
